# Oral presentations

**DOI:** 10.1054/bjoc.2001.1917

**Published:** 2001


*British Journal of Cancer* (2001) **85**(Suppl 1), 12–32
© 2001 Cancer Research Campaign
doi: 10.1054/ bjoc.2001.1917, available online at http://www.idealibrary.com on IDEAL®

### 1.1 FUNCTIONAL ANALYSIS OF THE CANDIDATE BLADDER TUMOUR SUPPRESSOR, DBCCR1

JH Gill*, E Pitt, H Nishiyama, N Hornigold, MA Knowles, ICRF Clinical Centre, St James University Hospital, Leeds, LS9 7TF, UK

Genomic alterations of chromosome 9q, particularly deletions, are the most common genetic alteration in all stages and grades of bladder cancer. *DBCCR1* is the only candidate tumour suppressor gene so far identified within the gene-poor critical region of deletion at 9q32–33. Previously, this gene was shown to be silenced by promoter hypermethylation in 50% of bladder cancer cell lines and be homozygously deleted in primary tumours, but small intragenic tumour-specific mutations have not been identified. Exogenous expression of DBCCR1 protein resulted in suppression of proliferation in NIH/3T3 cells, due to an accumulation of cells within $G_1$ of the cell cycle, further supporting the hypothesis that *DBCCR1* is a tumour suppressor gene. Here we sought to further these observations, and to provide a role for DBCCR1 in the bladder. Expression of DBCCR1 in bladder cancer cell lines resulted in in vitro growth inhibition, reduced in vitro colony formation and soft agar clonogenicity and a suppression of tumorigenicity in an in vivo mouse model. Furthermore, since DBCCR1 shows no homology to any known protein, the presence of functional protein domains within DBCCR1 was also investigated. DBCCR1 was found to be located primarily cytoplasmically and to contain three main functional regions. The involvement of these regions is currently under investigation. Taken together these observations reinforce a role for DBCCR1 in growth control, and support the hypothesis that this is the tumour suppressor gene targeted by 9q32–33 deletion in bladder cancer.

### 1.2 INTERLEUKIN-10 GENE POLYMORPHISMS INFLUENCE TUMOUR DEVELOPMENT IN CUTANEOUS MALIGNANT MELANOMA

WM Howell[1], SJ Turner[1], AC Bateman[2], JM Theaker[2], [1]Human Genetics, University of Southampton, [2]Department of Histopathology, Southampton University Hospitals, Southampton SO16 6YD, UK

Cutaneous malignant melanoma (CMM) is a serious and often fatal malignancy, in which patients may develop an anti-tumour immune response. Conflicting evidence suggests that IL-10 contributes to tumour escape from the immune response, but can also have an anti-tumour effect, via inhibition of angiogenesis. To distinguish between these models and to determine whether genotypes associated with differential IL-10 expression confer susceptibility to and/or influence prognosis in CMM, 153 British caucasian CMM patients and 158 controls were genotyped for IL-10 promoter SNPs by ARMS-PCR. The IL-10 -1082 AA low expression genotype was increased in incidence among CMM patients (26.8% vs 17.1%; $P$ = 0.04; OR = 1.8 (95% CI 1.0–3.1)). In addition, IL-10 genotypes showed significant associations with three of four prognostic indicators examined:-IL-10 -1082 GG and -1082, -819 and -592 GCC/GCC compound high expression genotypes were associated with horizontal (non-invasive) vs vertical (invasive) tumour growth (38.3% vs 20.4%; $P$ = 0.02; OR = 2.4 (0.4–1.0) and 37.8% vs 20.8%; $P$ = 0.03; OR = 2.3 (1.1–5.0) respectively); the IL-10 -1082 AA low expression genotype was associated with more advanced (Stage II–IV vs Stage I) disease (34.5% vs 19.0%; $P$ = 0.04; OR = 2.2 (1.0–4.8)); Finally, the IL-10 -1082 AA and -1082, -819 and -592 ACC/ACC, ACC/ATA and ATA/ATA compound low expression genotypes were significantly increased in frequency among patients with thicker (> 1.5 mm) primary vertical growth phase tumours (20/50 (40.0%)) vs 8/52 (15.4%); $P$ = 0.005; OR = 3.7 (1.4–9.4) and 18/47 (38.3%) vs 7/48 (14.6%); $P$ = 0.009; OR = 3.6 (1.4–9.8) respectively). Also, tumour thickness was significantly greater among patients with low IL-10 vs high IL-10 expression genotypes (2.72 mm ± 3.32 vs 1.16 mm ± 1.11; $P$ = 0.002).

These results indicate that genotypes associated with high levels of IL-10 expression in vitro are protective in CMM, while low expression genotypes are a risk factor for more advanced/poorer prognosis disease and may confer susceptibility to CMM. Although the influence of IL-10 on melanoma development is likely to be complex, these results support recent findings that IL-10 has an anti-tumour effect in CMM, possibly via inhibition of angiogenesis. These findings may have implications for tumour immunotherapy.

### 1.3 THE INHIBITOR OF APOPTOSIS GENE *SURVIVIN* IS UPREGULATED IN OESOPHAGEAL CANCER

DM Beardsmore, C Verbeke, AI Sarela, AGK Li, CL Davies, PJ Guillou, GWB Clark, Academic Department of Surgery and Histopathology, St James's University Hospital, Leeds, LS9 7TF, UK

**Background** *Survivin* is a recently described Inhibitor of Apoptosis gene and its expression in cancer has been shown to alter tumour behaviour. Consequently, *Survivin* has been proposed as a potential therapeutic target and prognostic molecular marker. We aimed to investigate the expression of *Survivin* mRNA and protein in oesophageal tumours.

**Methods** *Survivin* mRNA was evaluated by RT-PCR on RNA extracted from 68 snap frozen oesophageal tumours and matched normal oesophageal mucosa. The *Survivin* PCR products were semi-quantitatively scored (0–4) after standardising with the expression of the control gene, G3PDH. Tissue was sampled from 41 resection specimens and 27 endoscopic biopsies comprising 56 (82%) adenocarcinomas [Type 1 = 32 (57%), Type 2 = 12 (21.5%) and Type 3 = 12 (21.5%)], 10 (15%) squamous cell cancers and 2 (3%) of mixed histology. 55% of tumours were poorly differentiated, 38% moderately differentiated and 7% well differentiated. Resection specimens were staged T1 = 5 (12%), T2 = 9 (22%), T3 = 27 (66%) and 30 were positive for nodal metastases. Immunohistochemistry was performed on a subset of 28 tumours using the polyclonal antibody SURV11A. Immunostaining was categorized by the percentage of tumour cells immunostained (0 = <5%, 1 = 5–25%, 2 = 26–50%, 3 = 51–75%, 4 = >75%) and the intensity of staining (1+, 2+ and 3+) and a Weighted Index (WI) was calculated.

**Results** By RT-PCR, 64 (94%) of oesophageal cancers and 53 (78%) of normal oesophageal mucosa samples were positive for mRNA expression. Using semi-quantitative scoring 37 (54%) of tumours showed upregulation of *Survivin* mRNA expression 27 (40%) similar expression and 4 (6%) relatively reduced expression compared to matched normal controls. Up-regulation of *Survivin* did not correlate with the clinicopathological variables of tumour histology, stage, differentiation, presence of nodal metastases, age and sex. The WI score revealed heterogenous protein expression for Survivin between the tumours with 13 (46%) showing relatively high levels of immunostaining (WI > 9), 7 (25%) moderate staining (WI 4–8) and 8 (29%) weak staining (WI < 4). In addition normal oesophageal mucosa stained positively and this was restricted to the basal layer of the mucosa.

**Conclusion** *Survivin* mRNA appears to be constitutively expressed at low levels in normal oesophageal mucosa and oesophageal cancer. However, 54% of these tumours show significant upregulation of *Survivin* mRNA expression. *Survivin* protein was also detected in these tumours using immunohistochemistry. There was considerable variability in the degree of staining between tumours and this differential expression of *Survivin* may potentially alter response to treatment.

### 1.4 THE NUCLEAR DEAD BOX PROTEIN P68 IS OVER-EXPRESSED AND POST-TRANSLATIONALLY MODIFIED IN COLORECTAL TUMOURS

RG Hislop, M Causevic, NM Kernohan, FC Carey, F Fuller-Pace, Dept of Molecular and Cellular Pathology, University of Dundee, Ninewells Medical School, Dundee, DD1 9TS, UK

The nuclear protein p68 is a prototypic member of a family of RNA helicases containing eight conserved motifs including the DEAD box (Asp-Glu-Ala-Asp). p68 expression is growth and developmentally regulated and appears to correlate with organ differentiation/maturation in the foetus. As other members of the DEAD box family are known to be over-expressed in tumour cell lines, we have compared p68 expression in normal colon, colorectal adenoma and colorectal carcinoma tissue.

Immunohistochemical staining of colon tissue sections showed increased levels of p68 in cancers compared to matched normal colon from the same patient. Western blotting also indicated a higher level of p68 expression in tumour tissue but while a single band was detected in most normal tissue extracts, p68 migrated as multiple forms with lower electrophoretic mobility in tumour tissue. The normal band was of lower intensity or completely absent in tumour tissue. These results were consistent among a preliminary sample of 20 patients. We observed no obvious over-expression of p68 at the RNA level. Transfection of cell lines with p68 and ubiquitin expression plasmids has shown that this phenomenon can be reproduced in tissue culture cells and provides a model for further detailed studies.

The results of this study suggest that there is an increase in the level of p68 in pre-invasive and invasive colorectal tumours. It is possible that accumulation of p68 occurs due to a fault in degradation of ubiquitinated proteins. We are currently investigating by RT-PCR whether there are underlying mutations in the p68 gene.

### 1.5 GENETIC ANALYSIS OF GASTRO-OESOPHAGEAL MALIGNANCY BY CGH

SC Stocks[1,2], FA Carey[2], AM Thompson[3], D Johnston[3], NM Kernohan[2], NR Pratt[2], Departments of [1]Human Genetics, [2]Molecular and Cellular Pathology and [3]Surgery, Ninewells Hospital and Medical School, Dundee DD1 9SY, UK

Gastro-oesophageal cancers are common throughout the world but because of late presentation are associated with a poor prognosis. World-wide, squamous cell carcinomas (SCC) are by far the most common form of oesophageal malignancy. However, in the West there has been a remarkable change in the epidemiology such that the UK rate of gastro-oesophageal adenocarcinoma (GOA) is among the highest in the world. We have used comparative genomic hybridisation (CGH) to investigate patterns of large scale genetic change in a series of approximately 100 gastro-oesophageal cancers. This unselected study has revealed distinct patterns of genetic change (gain and loss) in SCC and GOA. Both tumour types showed frequent gain of 3q, 5p, 8q, 20q and loss of 18q. However, SCC showed specific amplification of 11q13 (cyclin D1) loss of 3p but seldom had whole arm gains of 13q or loss of 17p (p53) whereas GOA had frequent gains of 13q and loss of 17p but rarely exhibited 3p deletion or 11q13 gain. The identification of consistent regions of abnormality for each tumour type implicates the effects of clonal karyotypic abnormalities in the pathogenesis of each type of malignancy. Furthermore, this study emphasises a role for CGH and related whole genome analysis techniques in characterising distinct tumour subtypes.

### 1.6 MORPHOMETRIC ANALYSIS OF HEAT SHOCK PROTEIN 27 EXPRESSION; A NOVEL MARKER OF INCREASED BREAST CANCER RISK

A Shaaban[†1], P O'Neill[2], A Dodson[1], CS Foster[1], [1]Department of Cellular and Molecular Pathology, Royal Liverpool University Hospital, Liverpool, [2]Clatterbridge Centre for Oncology, Merseyside, UK

Heat shock proteins (hsps) are molecular chaperones that are induced in cells in response to different stimuli. In previous studies, hsp27 overexpression was found to correlate with poor prognosis in breast carcinomas. However, its role in precancerous breast lesions has not yet been determined. Dysregulation of its expression in precancerous breast lesions may represent an early step in mammary oncogenesis. Therefore, we conducted a case-control study on paraffin embedded biopsy specimens from patients who subsequently developed breast cancer (cases, $n$ = 120) against controls, age and date of biopsy matched, ($n$ = 382) who did not develop breast cancer spanning a twenty year follow-up period. Foci of hyperplasia of thr usual type (HUT) were identified in tissues and the relative risk of HUT was defined. Tissue sections containing foci of HUT ($n$ = 162) and surrounding normal lobules ($n$ = 93) from cases ($n$ = 28) and controls ($n$ = 21) were stained using a monoclonal antibody (Novocastra Laboratories Ltd.) for hsp27 with heat pretreatment for antigen unmasking. The percentage of positive cells was assessed and the mean area and optical density (OD) of positive staining in hyperplastic and normal foci were quantified using morphometric image analysis. The mean expression in HUT (±SD) was 29.32% (29.45) in biopsies from patients who subsequently developed breast cancer compared with 13.92% (18.67) in controls. This difference was highly significant ($P < 0.001$).Among cases subsequently developing breast cancer, a significant overexpression of hsp27 was found in HUT foci compared with normal lobules ($P < 0.0001$). In HUT , there was a strong positive correlation between mean hsp27 expression and densitometry (OD) (r = 0.836, $P < 0.0001$). The latter was higher in cases compared with controls. The mean HUT OD (±SD) was 0.49 (0.21) in cases developing breast cancer and 0.43 (0.25) in controls while normal foci showed OD of 0.42 (0.21) and 0.32 (0.23) in cases and controls respectively. Using a cut-off point for hut OD of 0.45, a significant difference was found between cases and controls ($P < 0.024$). These data suggest a previously undefined role of hsp27 during mammary carcinogenesis and indicate that the overexpression of hsp27 may define a subnet of hyperplastic breast lesions, which are phenotypically benign but biologically aggressive. This might have important implications for the improvement of screening and management regimens.

### 1.7 POLYMORPHISM IN GST P1 ASSOCIATED WITH RISK OF ACUTE MYELOID LEUKAEMIA FOLLOWING CHEMOTHERAPY

JM Allan[1], CP Wild[1], S Rollinson[2], EV Willett[1], GJ Dovey[2], A Moorman[1], E Roman[1], RA Cartwright[1] and GJ Morgan[2], [1]Epidemiology and HSR and [2]Department of Haematology, School of Medicine, University of Leeds

Glutathione *S*-transferases (GSTs) detoxify potentially mutagenic and cytotoxic DNA-reactive electrophiles by conjugation to glutathione. In addition to protecting against endogenously-formed and environmentally-derived electrophiles, GSTs, and particularly GST P1, also protect against the cytotoxic effects of several chemotherapeutic agents. Ironically, these agents, which include cyclophosphamide, etoposide, adriamycin and cisplatin derivatives, are also suspect human mutagens and carcinogens, and therefore may confer a risk of cancer. Polymorphisms of functional significance exist in at least 3 genes encoding GSTs, including *GST M1*, *GST T1* and *GST P1*. We hypothesise therefore, that polymorphisms in these genes alter susceptibility to chemotherapy-induced carcinogenesis. Therapy-related acute myeloid leukaemia (t-AML) is a devastating complication of long-term cancer survival. Identification of genetic determinants may help to identify individuals at increased risk of developing t-AML. To this end, we have examined 89 cases of t-AML for the frequency of polymorphisms in *GST T1*, *GST M1* and *GST P1*, and compared this to the frequency in a matched control population. Gene deletion of *GST T1* was associated with susceptibility to t-AML in males (OR 2.61, 95% CI 1.11–6.09) but not in females (OR 1.06, 95% CI 0.46–2.47). Gene deletion of *GST M1* was not significantly associated with susceptibility to t-AML. Individuals with the *GST P1* codon 105 valine allele were significantly over-represented in cases compared to their matched controls, suggesting an increased risk of developing t-AML [Odds Ratio (OR) 2.26, 95% Confidence Interval (CI) 1.38–3.71, 89 cases, 259 controls]. Moreover, a significantly increased risk of t-AML for individuals with codon 105 valine was only seen in those with prior exposure to chemotherapy (OR 4.00, 95% CI 1.95–8.21, 51 cases, 150 controls), and particularly with prior exposure to a known GST P1 substrate (OR 6.56, 95% CI 2.11–20.43, 22 cases, 64 controls). *GST P1* codon 105 status was not associated with risk of t-AML in those individuals with prior exposure to radiotherapy alone (OR 1.18, 95% CI 0.57–2.44, 38 cases, 109 controls). Thus, inheritance of at least one valine allele at *GST P1* codon 105 confers a significantly increased risk of developing t-AML following cytotoxic chemotherapy, but not radiotherapy. This work was supported by the Kay Kendall Leukaemia Research Fund and the Leukaemia Research Fund.

### 1.8 PROTEOMIC IDENTIFICATION OF DIFFERENTIALLY EXPRESSED PROTEINS IN RENAL CELL CARCINOMA

RD Unwin[1], R Craven[1], P Harnden[1], DJ Pappin[2], N Totty[2], PJ Selby[1], RE Banks[1], [1]ICRF Cancer Medicine Research Unit, St. James University Hospital, Leeds, LS9 7TF, [2]ICRF Protein Sequencing Laboratory, 44 Lincolns Inn Fields, London, WC2A 3PX, UK

Renal cell carcinoma (RCC) is the tenth most common cancer worldwide and is responsible for around 5% of all cancer deaths. Approximately half of all RCC patients are diagnosed with advanced disease, with 5-year survival for rates of about 20%.

In order to identify potentially novel markers or drug targets for RCC, we have used two-dimensional gel electrophoresis and mass spectrometry to identify proteins that are differentially expressed by clear cell renal tumours when compared to normal kidney.

The protein profiles of matched tumour and normal tissues from 6 patients with grade 3 clear cell RCC were studied. Computer analysis revealed a total of 73 potential differentially expressed proteins, 32 of which are upregulated in 4/6 and 41 that appear to be downregulated in 6/6 RCCs. Proteins were identified by peptide mass fingerprinting and MALDI mass spectrometry. Some of the proteins identified have previously been shown to be overexpressed in RCC including pyruvate kinase and Mn-superoxide dismutase whilst some proteins have not previously been linked to tumorigenesis, such as annexin II, lamin B1 and cofilin.

A number of proteins identified by this screen are currently being further characterised using RT-PCR, tandem mass spectrometry and immunohistochemistry to ascertain their potential role in either disease pathogenesis or as potential disease markers.

### 2.1 DEVELOPMENT OF MOLECULAR IMAGING PARADIGMS

EO Aboagye, CRC PET Oncology group, Department of Cancer Medicine, Imperial College School of Medicine, Du Cane Road, London W12 0NN, UK

Imaging probes and paradigms can be used to enhance the scientific impact of cancer trials. This abstract will focus on the use of imaging to measure hypoxia, proliferation and enzyme function.

Hypoxia occurs to a variable extent in tumours and is an important determinant of therapeutic response and survival. We have developed SR 4554 as a magnetic resonance spectroscopy (MRS)-compatible probe for the measurement of hypoxia. The design features of SR 4554 were consistent with its in vivo pharmacokinetics. Proof that retention of SR 4554 is hypoxia-dependent was provided by its enzymology, subcellular distribution in spheroids and tumour retention. Differential retention was demonstrated in tumours with different radiobiological hypoxic fraction and following modulation by carbogen and hydralazine. Based on its interesting properties, SR 4554 has been selected for clinical development and is now in Phase 1 trials.

There is the need to develop new assays, which can be used to evaluate novel mechanism-based cytostatic agents in patients. Towards this end, we are developing 2-[$^{11}$C]thymidine and 2′-[$^{18}$F]fluorothymidine for measuring antiproliferative activity by positron emission tomography (PET). Proof that these probes can measure the inhibition of proliferation in the absence of tumour shrinkage has been provided for trichostatin-A in HT29 tumour bearing mice. The relationship between inhibition of proliferation and specific effects of the drug including inhibition of histone deacetylase and histone H4 hyperacetylation has been studied. Clinical validation of 2-[$^{11}$C]thymidine has also been performed. The retention of 2-[$^{11}$C]thymidine was shown to correlate with MIB 1 index in gastrointestinal cancers of patients.

Dihydropyrimidine dehydrogenase (DPD) is the proximal and rate limiting enzyme in the catabolism of 5-fluorouracil. Large variations in enzyme expression occur and have been shown to influence the pharmacodynamics of 5-fluorouracil. Eniluracil is a mechanism-based inactivator of DPD. Proof that eniluracil acts in a predictable manner in patients was provided by studying the in vivo pharmacokinetics of 5-[$^{18}$F]fluorouracil (5-[$^{18}$F]FU). The rapid conversion of 5-[$^{18}$F]FU by normal liver (the organ with the highest DPD activity) to [$^{18}$F]fluoro-β-alanine, as well as the hepatobiliary excretion of [$^{18}$F]fluoro-β-alanine bile conjugates were inhibited by eniluracil. In the tumours of these patients, the reduction in catabolism led to a significant increase in tumour 5-[$^{18}$F]FU+anabolite levels. Knowledge obtained from these studies has been used to assess existing and novel biomodulators of 5-fluorouracil.

### 2.2 OPTIMISATION OF REDUCTASE ENZYMES FOR USE IN HYPOXIA REGULATED GENE DIRECTED ENZYME PRODRUG THERAPY

RL Cowen[1], KJ Williams[1], AV Patterson[2], SK Robinson[1], F Sheppard[1], BA Telfer[1], L-Y Lian[3], IJ Stratford[1], [1]School of Pharmacy, University of Manchester, Manchester, M13 9PL, UK, [2]Auckland Cancer Society Research Centre, Faculty of Medical and Health Sciences, University of Auckland, Private Bag 92019, New Zealand, [3]Department of Biomolecular Sciences, University of Manchester Institute of Science and Technology, Manchester M60 IQD, UK

Regions of low oxygen tension (hypoxia), which exist within most solid tumours, can be exploited as a tumour specific condition leading to the development of bioreductive drugs, which are specifically activated to become cytotoxic within hypoxic cells. Here we present a gene therapy strategy that exploits hypoxia in tumours using it as a tumour specific trigger to achieve overexpression of reductase enzymes. These enzymes can donate single electrons ($1e^-$) and can thereby activate any prodrug with an appropriate $1e^-$ reduction potential. This will increase the efficacy of bioreductive agents, the activation of which will be tightly controlled by hypoxia at both a transcriptional and metabolic level.

When cells become hypoxic, a tissue stress response is activated to increase expression of genes involved in energy metabolism and angiogenesis. Activation of these hypoxically inducible genes involves *cis*-acting hypoxia responsive elements (HRE). By introduction of HRE sequences within the promoter region of a therapeutic transgene cassette it is possible to hypoxically regulate the expression of the transgene. We have generated stable HT1080 human fibrosarcoma cell lines transfected with a bicistronic cassette encoding for human cytochrome P450 reductase (P450R) and green fluorescent protein (clone R9), or GFP reporter alone (clone GFP5), under the transcriptional regulation of the HRE derived from the phosphoglycerate kinase 1 gene (PGK-1). When grown as tumour xenografts in nude mice, the R9 and GFP-5 cells show similar growth rates and response to radiotherapy with a single dose of 10 Gy. Combining radiotherapy with administration of the bioreductive drug, RB6145 had no effect on the efficacy of 10 Gy in the GFP-5 tumours, but led to a 50% cure rate (tumour free for 100 days following therapy) in the R9 group.

We are now evaluating the use of recombinant adenoviral vectors to achieve the high levels of tumour specific P450R expression that will be required in an effective therapy. We have also identified an alternative HRE sequence from lactate dehydrogenase A which will provide us with increased hypoxia responsive gene expression and most recently we are investigating ways of optimising the reductase enzyme itself by retargeting the enzyme to a different subcellular compartment.

### 2.3 INTEGRATING THE TRANSCRIPTIONAL RESPONSE TO HYPOXIA AND IONIZING RADIATION – APPLICATIONS FOR GENE THERAPY

S Robinson, A Patterson, N Chadderton, K Williams, R Cowen, I Stratford, Experimental Oncology Group, School of Pharmacy and Pharmaceutical Sciences, University of Manchester, Oxford Road, Manchester M13 9PL, UK

Neoplastic cells sense hypoxia and respond by altering the expression of a variety of genes, primarily through a HIF-1 dependent *trans*-activation of hypoxia responsive elements (HREs). HREs have been employed to transcriptionally target gene expression to hypoxic tumours. Rather than just optimising the hypoxia-responsiveness of potential chimaeric promoters, we have attempted to develop dual hypoxia and X-ray inducible promoters, such that the combination of both stimuli might provide a greater-than-additive effect upon transcriptional output.

A number of early response genes are induced in response to ionising irradiation (e.g. c-fos, egr-1, UpA). This response is known to be dependent upon the presence of serum response elements (SREs or CarGs; CC[A/T]$^6$ GG) within the promoter regions. A chimaeric promoter was constructed, composed of the short X-ray responsive Egr-1 promoter (425 b.p.) fused to a pentamer of the Epo gene minimal HRE (140 b.p.). The promoter was introduced into the pGL3 basic luciferase reporter vector. A number of HRE-driven luciferase constructs were also made. Each vector was transiently transfected into a panel of human carcinoma cell lines and their response to either hypoxia (1% $O_2$), X-rays (5 Gy), or both stimuli, were tested.

In vitro the pGL3.egr-1 plasmid was responsive to 5 Gy, the pGL3.Epo.egr-1 vector was responsive to both stimuli, with the combination of hypoxia and X-rays potentiating the expression of luciferase. A response to X-rays as well as hypoxia was seen in the HT1080 cell line but not in the MDA468s. Further improvements to the chimaeric promoter were explored by utilising the minimal CarG elements (juxtaposed by the ELK-1 binding sites), preliminary results suggest that their response may also be cell line dependent. This data demonstrates that it is possible to create a ubiquitous X-ray and hypoxia responsive chimaeric promoter.

### 2.4 MANIPULATION OF P450 GENE EXPRESSION IN TUMOURS; A NOVEL APPROACH FOR TARGETED ACTIVATION OF BIOREDUCTIVE PRODRUGS

SR McKeown, CM Hughes, G Keilty, HO McCarthy, M Murray, DG Hirst, T Robson, School of Biomedical Sciences, University of Ulster, Jordanstown, N. Ireland, UK

We are developing a gene-directed enzyme prodrug therapy (GDEPT) strategy to enhance the metabolism of a novel bioreductive drug, AQ4N. Bioreductive drugs are metabolically activated in the hypoxic cell environment allowing effective targeting of hypoxic radioresistant tumour regions. We aim to achieve additional layers of selectivity by using an X-ray inducible promoter linked to our therapeutic gene (cytochrome P450s). This strategy would enhance metabolism of the drug only within the radiation field. Furthermore, normal tissue would be unaffected as the bioreductive drug is only activated in hypoxic conditions. We have identified human cytochrome P4501A1 (CYP1A1) to be the major prodrug activating enzyme by in vitro drug metabolism studies of AQ4N using a range of human supersomes (Gentest). Further to these studies, RIF1 murine tumour cells transfected with CYP1A1 cDNA displayed greatest DNA damage and clonogenic cell kill following treatment with AQ4N under hypoxia. We are presently testing the ability of these transfectants to enhance anti-tumour effectiveness of AQ4N in combination with radiation in vivo. In addition, we have observed a dose dependent increase of the GFP reporter gene using the X-ray inducible WAF1 promoter. GFP was induced 1.9, 3.1 and 4.2 fold under the control of the WAF-1 promoter by doses of 2, 4 and 6 Gy X-rays respectively. Interestingly, this promoter was also induced by acute hypoxia (2 h). We now aim to link this promoter to CYP1A1 for selective activation in-vivo.

### 2.5 HORSERADISH PEROXIDASE AND INDOLE-3-ACETIC ACID FOR HYPOXIA- AND RADIATION-REGULATED GENE THERAPY OF CANCER

O Greco*, GM Tozer, LK Folkes, P Wardman, SD Scott, B Marples, M Joiner, GU Dachs, Gray Laboratory CRT, Mount Vernon Hospital, Northwood HA6 2JR, UK

The plant enzyme horseradish peroxidase (HRP) and the non-toxic plant hormone indole-3-acetic acid (IAA) represent a novel combination for gene-directed enzyme/prodrug therapy of cancer (GDEPT, Greco et al, Cancer Gene Ther 7: 1414, 2000). Transfection with the HRP cDNA followed by incubation with IAA induced selective toxicity in a panel of cell lines of human origin. Prodrug activation was fast and efficient. Significant cytotoxicity was induced after only 2-hours' exposure, which was further increased after 24-h. In transient HRP-transfectants, up to 3-log cell kill was induced at doses of IAA non-toxic to mock transfectants expressing the marker GFP. The HRP/IAA system was similarly effective in the tumour conditions of anoxia and in hypoxia (0.1% $O_2$), although different mechanisms of cytotoxicity appear to be involved. A strong bystander effect was induced, since ~70% the population exposed to IAA in air or hypoxia could be killed when only 5% of the cells expressed the HRP. Conditioned-medium switch experiments showed that the toxic metabolite is a long-lived species able to cross cell membranes, and that cell contact is not required for bystander killing. When compared to the well-established system HSV TK/GCV, the HRP/IAA combination showed in T24 bladder carcinoma cells increased efficacy and selectivity in vitro, both in oxic and anoxic conditions. The interaction of HRP-mediated GDEPT with ionising radiation was evaluated. After pre-incubation with IAA a marked increased in sensitivity to X-rays was selectively induced in HRP-expressing T24 cells. Sensitisation enhancement ratios (SERs) of 1.8 (0.1 mM IAA) and 3.1 (0.5 mM IAA) were measured. No significant difference in the response to radiation was observed in $HRP^-$ cells in the presence of the prodrug. Activated IAA has previously been observed to react with DNA (Folkes et al, Biochem Pharmacol. 57: 375, 1999) and to deplete glutathione, which could lead to sensitisation to radiation.

To specifically target the radio-and chemoresistant population in solid tumours, the HRP gene was placed under the control of hypoxia and/or radiation responsive promoters. Five copies of hypoxia responsive elements (HREs) from the *PGK-1* or the *EPO* gene were inserted in the basal cytomegalovirus (bCMV) promoter. After 24 h-hypoxic incubation, the *EPO* and the *PGK-1* HREs induced a 30–40 fold and a 5–6 fold increase in HRP expression respectively. Selective HRP production in irradiated cells was achieved by using radiation responsive CArG elements (Marples et al, Gene Therapy 7: 511, 2000). After 5 Gy X-irradiation, a selective 2–3-fold increase in transgene expression was detected.

### 2.6 HYPOXIA-INDUCIBLE GENE EXPRESSION IN MACROPHAGES: ROLE OF HIFs- 1 AND -2, AND USE OF cDNA ARRAYS TO IDENTIFY UP-REGULATED GENES

B Burke, D Gill, M Wells, CE Lewis, Tumour Targeting Group, Section of Pathology, Division of Genomic Medicine, University of Sheffield Medical School, Sheffield S10 2RX, UK

Hypoxia is a common feature of solid tumours. Macrophages migrate continually into tumours from the bloodstream, and congregate in large numbers in hypoxic sites, playing an important part in stimulating tumour angiogenesis. However, the effects of hypoxia on gene expression have not been characterised in human macrophages. In this study we have first determined the effect of hypoxia on the level of two related transcription factors, hypoxia-inducible factor -1 and -2 (HIFs-1 and -2) in primary human macrophages in vitro. We then used cDNA array hybridisation to identify hypoxia-induced changes in the level of mRNA for 1205 different genes in macrophages. We show that, contrary to previous reports, hypoxic human macrophages produce abundant HIF-1 (in vitro and in various types of human tumours), and that although they also produce HIF-2, this is less abundant than HIF-1. We also show that exposure of primary macrophages to 0.5% oxygen increased mRNA levels for a number of genes including matrix metalloproteinase 7 (MMP 7 or matrilysin), vascular endothelial growth factor (VEGF), erythrocyte glucose transporter 1, and EGF response factor 1 (ERF1). The promoters of a number of these hypoxia-regulated genes contain hypoxia-responsive elements (HREs), short DNA sequences known to bind HIFs, leading to enhanced expression of the associated genes. As the HREs of these genes are clearly active in hypoxic macrophages, they have the potential to be used in a novel gene therapy which would employ macrophages to carry hypoxia-activated therapeutic genes into hypoxic tumour sites. This approach could also have utility in the treatment of other diseases in which macrophages accumulate in hypoxic/ischemic tissues (e.g. in joints affected by rheumatoid arthritis).

### 2.7 THYMIDINE PHOSPHORYLASE INDUCES CARCINOMA CELL OXIDATIVE STRESS AND PROMOTES SECRETION OF ANGIOGENIC FACTORS

NS Brown[1], A Jones[2,3], C Fujiyama[2], AL Harris[2], R Bicknell[1], [1]Molecular Angiogenesis Lab, and [2]Molecular Oncology Lab, ICRF, WIMM, JR, Hospital, Oxford, UK, [3]Department of Urology, Churchill Hospital, Oxford, UK

**Introduction** Thymidine phosphorylase (TP) is a potent angiogenic factor that correlates with poor prognosis in a wide variety of tumours (1). TP is not secreted by the carcinoma cell, and its angiogenic activity is known to be dependent upon the catabolism of thymidine to thymine and 2-deoxy-D-ribose-1-phosphate (2dDR1P) (2). It has remained unclear why this reaction stimulates new vessel growth. We have studied transfected bladder carcinoma cell lines to determine the mechanism by which TP causes angiogenesis.

**Methods** RT112 cells were transfected with full-length human TP cDNA to give the cell line RT112-TP, and were transfected with an empty vector to give the control cell line RT112-EV. The cells were then exposed to 200 µM thymidine for 16 hours, leading to high levels of thymidine catabolism in RT112-TP.

**Results** When exposed to thymidine, the oxidative stress marker haem oxygenase-1 was induced 4-fold within RT112-TP, but was not significantly induced within RT112-EV. The increase in HO-1 was blocked by both antioxidants and by excess thymine. Thymidine catabolism by TP therefore induces carcinoma cell oxidative stress. In these oxidatively stressed carcinoma cells, secretion of the angiogenic factor vascular endothelial growth factor was increased 2-fold, production of interleukin-8 went up 6-fold, and matrix metalloproteinase-1 levels were elevated 1.5-fold (3).

**Conclusions** It appears that TP promotes angiogenesis by inducing carcinoma cell oxidative stress. When TP overexpressing carcinoma cells experience oxidative stress they increase their production of the secreted angiogenic factors VEGF and IL-8. These will act directly upon endothelial cells to cause vascularisation of the tumour.

1. N.S Brown & R. Bicknell, (1998) *Biochem J* **334**: 1–8 (review)
2. A. Moghaddam. et al (1995) *Proc Natl Acad Sci* USA **92**: 998
3. N.S. Brown et al(2000) *Cancer Res* **60**: 6298–6302

### 3.1 PHASE III TRIAL OF DOSE ESCALATION USING CONFORMAL RADIOTHERAPY IN PROSTATE CANCER: SIDE EFFECTS AND PSA CONTROL

D Dearnaley[1], E Hall[1], C Jackson[1], D Lawrence[1], R Huddart[1], R Eeles[1], J Gadd[2], A Warrington[2], M Bidmead[2], A Horwich[1] [1]Institute of Cancer Research/[2]Royal Marsden NHS Trust, Sutton & London

**Background** This prospective randomised phase III single centre trial was designed to evaluate the side effects and efficacy of dose escalation using CFRT and in a 2×2 factorial design to study the appropriate radiotherapy planning 'safety margin'.

**Patients and Methods** 126 men with localised prostate cancer were treated with initial (3–6 months) androgen suppression and then randomised between (1) standard dose (SD) CFRT treatment (64 Gy) or standard treatment with high dose (HD) boost (74 Gy) and (2) Phase I margin of 1.0 cm (M1.0) or 1.5 cm (M1.5). The boost (10Gy) was delivered to the prostate only with no margin. Patients were stratified according to risk of seminal vesicle involvement (SV+) and treatment volumes defined appropriately. Patient and tumour characteristics were evenly balanced between the groups with median age of 67 years, presenting PSA of 15 ng/ml and low/moderate risk of SV + of 29%/71%. Study endpoints included acute toxicity (RTOG), late side effects (RTOG/LENT-SOM), biochemical (PSA) control, 2 year post-treatment biopsies, clinical disease control and overall survival.

**Results** Median follow-up after CFRT was 3.5 years (range 0.6–5.2 years). *Acute side-effects*: There was no observed difference in bowel side effects between randomised groups, but bladder side effects were more common both during and after treatment in M1.5 compared with M1.0 group ($P$ = 0.002), and after treatment in the HD compared with SD group ($P$ = 0.006). *Late side-effects*: For all patients bowel or bladder RTOG ≥ 3 toxicity was uncommon (bowel 2%, bladder 5%). Bowel side-effects (RTOG ≥ 2) were seen in 23%/11% of HD/SD groups respectively ($P$ = 0.06) and 21%/13% of the M1.5/M1.0 groups respectively ($P$ = 0.2), but all three men with grade ≥3 side effects were in the HD M1.5 group. Bladder side-effects (RTOG ≥ 2) were recorded in 18%/13% of HD/SD groups ($P$ = 0.3) and 21%/10% of the M1.5/M1.0 groups ($P$ =.07) respectively. A similar rate of impotence (59%) was seen in all groups.

**Biochemical control** PSA levels were consistently ≤2ng/ml in 82% and 69% of HD and SD group after completion of treatment ($P$ = 0.06). Control rates were 75% and 76% for M1.0 and M1.5 groups.

**Comment** These results suggest dose escalated CFRT can be given with an acceptable level of acute/late toxicity, but volume and dose effects are detectable for bladder and bowel side effects. Early follow-up data suggests a possible improvement in biochemical control with increased dose, but there was no evidence to suggest any advantage for the larger radiotherapy margin. The results have been used to inform the MRC Trial RT01 Data Monitoring Committee and will be combined in meta-analysis with the national trial.

### 3.2 PROSTATE BRACHYTHERAPY; A PROSPECTIVE ANALYSIS OF UROLOGICAL SYMPTOMS.

Declan Cahill. Stephen Langley, Abdul Ismail, Robert Laing. St. Lukes Cancer Centre, Royal Surrey Hospital, Guildford GU2 5XX

Prostate brachytherapy is becoming an accepted treatment for organ confined prostate cancer, and 12-year PSA free survival rates are equivalent to those achieved with surgery. However, increased urinary symptoms occur in a significant proportion in the first year. Data on urinary symptoms have been collected prospectively on the 71 patients treated with permanent iodine implants These data include formal uro-dynamic measurements (UDS) pre-treatment in a sub-group.

1) Do baseline assessments or dosimetry predict symptom score after treatment?
2) Can pre-treatment uro-dynamics determine who will go into retention?

**Methods** A descriptive cohort analysis. Baseline assessments collected were:

1) IPSS (prostate international symptom score) graded mild 1–7: moderate 8–19: severe 20–35
2) prostate volume according to ultrasound
3) urodynamics categorised as stable or unstable: obstructed or non-obstructed or equivocal

Dosimetry was expressed as D90 (the dose delivered to 90% of the prostate). V100 (volume of prostate receiving 100% of the prescribed dose) and V150 (150%).
Outcome measures were IPPS at 6, 12, 24, 39 weeks, and the need for intermittent self-catheterisation (ISC)

**Results** Of the 71, 12 were excluded from the analysis, as baseline data were incomplete at this time.

| | Mean IPSS (95% Confidence interval) by weeks since treatment | | | | |
|---|---|---|---|---|---|
| | 0 | 6 | 12 | 26 | 39 |
| Cohort | 7.7<br>(6.3–91) | 20.8<br>(17.7–23.7) | 15.8<br>(13.2–18.5) | 15.5<br>(12.6–18.7) | 12.1<br>(7.9–16.3) |
| Mild | 3.3<br>(2.4–4.1) | 18.2<br>(14.0–22.4) | 13<br>(9.9–16.1) | 12.6<br>(7.1–18.2) | 7.3<br>(2.8–11.8) |
| Mod/sev | 11.9<br>(10.4–13.5) | 23.5<br>(19.4–27.7) | 18.5<br>(14.5–22.1) | 17.1<br>(14.1–1.25) | 15.9<br>(9.8–8.22) |
| *P** | <0.001 | 0.032 | 0.015 | 0.052 | 0.012 |
| N | 59 | 35 | 31 | 28 | 16 |

*Two-sample t test with equal variances

**3.2 Cont'd**

Those patients with mod/severe IPPS pre-treatment had significantly worse symptoms on follow up.

There were no differences in IPSS in those receiving external beam as well as brachytherapy compared to brachytherapy alone.

Urodynamic data have been collected on 33 individuals of whom 19 were obstructed. 8 equivocal and 6 unobstructed. To ease symptoms 14/71 required ISC for a short time of whom none were in the unobstructed group. 1 patient, who was in the obstructed group, required TURP.

Neither post implant dosimetry or prostate volume significantly predicted outcome.

**Conclusion** In this pilot study we have shown that patients' symptoms increase following brachytherapy and the degree of symptoms are related to the initial symptom score and obstruction on UDS. However at 39 weeks, the symptom scores are returning to baseline. The addition of external beam RT, prostate volume, V150 were not predictive of symptom score. Prostate brachytherapy remains a well-tolerated effective treatment for appropriately selected patients.

### 3.3 A PHASE II STUDY OF SYNCHRONOUS CHEMO-RADIOTHERAPY FOR LOCALLY ADVANCED BLADDER CANCER.

SA Hussain[1,2], DD Moffitt[1], JG Glaholm[2], D Peake[2], DMA Wallace[2], ND James[1,2] [1]CRC Institute for Cancer Studies, University Hospital Birmingham. [2]Queen Elizabeth Hospital, Edgbaston, Birmingham, UK

**Purpose** We have previously reported results of a phase I/II study of synchronous chemo radiotherapy with 5-fluorouracil (5-FU) and Mitomycin-C (MMC) in patients with muscle invasive bladder cancer. We now report updated results from our ongoing phase II trial with the optimised regimen.

**Method** Patients with T2-T4 N0/Nx M0 muscle invasive bladder cancer were entered into this single centre study. Patients received 55 Gy/20 fractions/4 weeks of radiotherapy. Concurrent chemotherapy was given with MMC 12 mg/m$^2$ day 1 and 5-FU 500 mg/m$^2$/24 hours for 5 days during weeks one and four of radiotherapy. The primary end point for the study was pathological response rate at 3 months; secondary endpoints were toxicity, disease-free and overall survival and bladder preservation rates.

**Results** A total of 35 patients have entered the trial from March 1998 to January 2001 and are available for toxicity assessment at the censor date of 10th February 2001. Median age was 68 (range 38–79) years, 27 males and 8 females; 3 patients were node positive; T2 9 (25%), T3a 5 (14%), T3b 11 (32%), T4 10 (29%), TCC grade 2, 8 (23%) and grade 3 27 (77%); 16/35 patients had hydronephrosis. Toxicity was mild to moderate (NCI-CTG grade 3: thrombocytopenia 4/35; diarrhoea 4/35). 26 patients underwent local response assessment, 5 patients were not evaluated due to early metastatic spread though their were no clinical suggestion of bladder failure, 1 patient died from probably unrelated cardiac cause, 3 patients are not yet due cysto-scopic assessment. Pathological complete response was seen in 19/26 (73%) patients at 3 months. Estimated 12-month survival was 63%. Two patients have required salvage cystectomy and, of patients still alive, 18/20 retain a functioning bladder at median follow up of 14 months.

**Conclusion** Chemoradiotherapy with the 5-day schedule is feasible in the management of elderly patients. The regimen has encouraging activity with acceptable toxicity in relatively poor prognosis patients. A randomised trial using the 5-day schedule compared to radical radiotherapy alone with standard versus reduced volume radiotherapy has the MREC approval and is due for national launch in July 2001.

### 3.4 AN EVALUATION OF HUMAN SKIN EPIDERMAL CELLS FOR HYPER-RADIOSENSITIVITY

N Shah, MC Joiner, MI Saunders, Marie Curie Research Wing and Gray Laboratory Cancer Research Trust, Mount Vernon Hospital, Northwood, Middlesex, UK

**Introduction** The laboratory phenomenon of hyper-radiosensitivity (HRS) describes an excess of cell kill at doses below 1 Gy relative to that predicted by the linear quadratic model. Mathematical modelling for malignant glioma suggests a doubling or more in the therapeutic index if radiation doses are given at 0.5 Gy per fraction three times a day to a total dose of 73.5 Gy. The epidermal basal cell layer of human skin, chosen as a model of normal tissue, is studied to verify the clinical existence of HRS.

**Methods** Epidermal basal cell density (BCD) was assessed by manual counting after H&E fixation of skin samples obtained by punch biopsy. The BCD of unirradiated normal human skin from 3 volunteers was studied to assess baseline variability. Twenty four patients receiving radical radiotherapy to the pelvis had the skin dose over the lateral radiation portals modified to compare doses of 0.5 Gy vs 1.0 Gy or more. Radiotherapy doses at the epidermal basal cell level were verified using thermoluminescent dosimeters (TLD). Skin biopsies, from both sides, were obtained until completion of radiotherapy. The changes in BCD were compared using non linear regression analysis.

**Results** Normal unirradiated skin BCD demonstrates significant intrapatient and interpatient variation ($P < 0.0001$). Analysis of 24 patients demonstrate a 2.03 (95% CI 1.80–2.28) fold statistically significant reduction in BCD, termed the Enhancement Ratio (ER), in favour of the lower skin dose (0.48 Gy vs 1.22 Gy). For individual patients, a statistically significant BCD reduction in favour of the low dose side was demonstrated in 14 (58%) patients (ER range 1.60–7.07). A non significant ER was demonstrated in 10 (42%) patients.

**Conclusion** In spite of baseline BCD variability, these results suggest HRS at the epidermal basal cell layer of skin. Individual patient analyses suggest that this may not be a universal phenomenon but this is subject to statistical uncertainty. The assessment of other cell kinetic parameters will help determine the true magnitude of HRS in human skin.

### 3.5 TARGETED RADIOIMMUNOTHERAPY – NOVEL DELIVERY OF CORONARY ARTERY RADIATION

EC Sims[1], MEB Powell[2], MT Rothman[1,3], TD Warner[1], [1]Dept. of Cardiac, Vascular & Inflammation Research, William Harvey Research Institute, [2]Dept. of Clinical Oncology & [3]Dept. of Cardiology, Bart's & The London, Queen Mary's School of Medicine & Dentistry, London, UK

**Introduction** Coronary artery brachytherapy is the most exciting breakthrough in cardiology of the last decade. Currently used techniques necessitate irradiation immediately post angioplasty, which leaves little scope to investigate key radiobiological issues. These include defining the optimal dose and fractionation schedule, timing of radiation delivery and radioprotection.

By inserting an antigen-coated stent at the time of angioplasty, we have developed a new technique enabling targeted radiotherapy to be delivered after angioplasty using radiolabelled antibody. This innovation allows the important issues of dose scheduling to be evaluated and minimizes radiation exposure to the medical team.

**Method** 15 mm metal stents were incubated in biotin-BSA (expt. 1), digoxin-BSA (expt. 2) or PBS as control to adsorb antigen. Commercially available anti biotin antibody and anti digoxin Fab fragments were labelled with $^{125}I$ in an iodination suite (24–32 kBq/µg). Antigen-coated stents were placed individually in the aortae of heparinised Wistar rats using an angioplasty balloon and the aortae were repaired. Return and maintenance of blood flow was confirmed by Doppler ultrasound of the iliac vessels. Radiolabelled antibody was then injected intravenously and the animals were sacrificed after 2 hours. The stents were carefully dissected out and captured radioactivity was recorded in a gamma counter. Organ samples were counted simultaneously to assess potential toxicity of the treatment allowing antibody distribution and clearance to be estimated.

**Results** Groups quoted as mean +/– standard error. Uptake of radioantibody by antigen-coated stents was significantly greater than control with minimal uptake by uncoated stents. Clearance of radioantibody was related to molecular weight being quicker with smaller Fab fragments.

| Expt. | Antigen | *N* = | Counts/ minute | kBq | Antibody bound (ng/stent) | *P* value |
|---|---|---|---|---|---|---|
| 1 | biotin | 3 | 27271 | 0.46 | 14.1 +/– 1.8 | 0.002 |
| | PBS | 3 | 1071 | 0.02 | 0.6 +/– 0.2 | 0.002 |
| 2 | digoxin | 4 | 42108 | 0.70 | 29 +/– 1 | $<10^{-4}$ |
| | PBS | 4 | 1752 | 0.03 | 1.2 +/– 0.4 | $<10^{-4}$ |

**3.5 Cont'd**

**Conclusion** This is the first study to demonstrate successful delivery of radiation to stents after the angioplasty procedure. Future aims are to maximize antigen loading on stents and to develop an antibody–antigen pair with optimised pharmacokinetics. This exciting technique promises immense therapeutic benefit in humans.

### 3.6 A NATIONAL ONLINE AUDIT OF RADIOTHERAPY IN HEAD AND NECK CANCER

ND James[1], G Robertson[2], H Forbes[3], K Jones[3], B Cottier[3], [1]CRC Institute for Cancer Studies, Birmingham, [2]Beatson Oncology Centre, Glasgow, [3]National Cancer Services Analysis Team, Clatterbridge Centre for Oncology, Wirral

**Introduction** Radiotherapy practice in head and neck cancer was identified as a target for a national audit as there are published data on the effects of delays in treatment and gaps in therapy on survival and published guidelines on their management. We utilised a novel online (*www.canceruk.net/natcansat/audit.htm*) audit tool to rapidly collect data (summarised in an accompanying abstract).

**Method** An electronic form in 2 parts was distributed to 56 eligible radiotherapy centres. The first part examined the centre's policies for managing gaps in therapy, the second collected data on 50 consecutive patients treated in that centre. Data entry was facilitated and standardised by the use of 'drop-down' lists. Patient data were collected on: primary site; stage; date of decision to treat; date of start and end of radiotherapy; prescribed dose and fractionation, steps taken to ensure timely completion of radiotherapy.

**Results** 55 centres returned data on a total of 2620 patients (2.9:1 male:female), mean age 62.8, interquartile range 54–72 years. Average delay to starting RT was 40 days with only 6 centres having an average wait of < 28 days. Commonest fractionation schedules were 54–55 Gy/20 fractions, 60–66 Gy/30–33 fractions, and 50 Gy/15–16 fractions. An analysis of fractionation by primary site, stage and centre is underway. Treatment was completed within 2 days of target in 78% of cases; an analysis of treatment prolongation by primary site, stage, fractionation and centre will be presented. Seven centres had no policy for dealing with treatment interruptions. Overall, 1467 (55%) patients had 1 or more treatment interruptions (service days 47%; machine breakdown 14%; staff shortages 2%, toxicity 12%; compliance 7%; patient died on treatment 3%; public holiday 67%). Of patients whose treatment was interrupted, 56% still completed on time due to compensatory steps: other machine 37%; treat twice 17%; biological compensation 18%; weekend or public holiday 5%; no attempted compensation 32%.

**Conclusions** This dataset gives a unique snapshot of radiotherapy practice in the UK for head and neck cancer for 1999–2000. It also sheds light on significant variations in the quality of care and shows a widespread failure to meet national targets (for waiting times) or guidelines (for compensation for gaps), which has serious implications both for patient outcomes and for implementation of the National Cancer Plan.

### 3.7 CLINICAL OUTCOME OF A RANDOMISED TRIAL OF CT SIMULATION VERSUS CONVENTIONAL SIMULATION FOR PALLIATIVE TREATMENT OF ADVANCED LUNG CANCER

M McJury[2], S Pledge[3], P Fisher[3], G Brown[1], C Anthony[1], M Hatton[3], J Conway[2], MH Robinson[1], [1]YCR Department of Clinical Oncology, [2]Department of Radiotherapy Physics, [3]Department of Radiation Oncology – all at Weston Park Hospital, Sheffield S10 2SJ

**Introduction** 113 patients were entered into a randomised trial comparing the use of CT simulation and conventional simulation in the palliative treatment of advanced lung cancer. The use of CT simulation was associated with markedly reduced treatment volumes compared to conventional simulation. This is the final report of the study detailing the clinical outcomes.

**Patients and methods** Complete patient data was available for 78 patients. Clinical assessment of the symptomatology was made pre-treatment and one month after treatment. Patient diary cards were completed for one month post-treatment.

**Results** 50% of patients in each arm received 17 Gy in 2 fractions and the other 50% 36 Gy in 12 daily fractions. The WHO score and general condition improved in 43% of those treated using conventional simulation compared to 30% and 20% respectively, of those using CT simulation. The figures for cough were 36.7% vs 38.9%; sputum production 40% vs 35.7%; haemoptosis 91.7% vs 100%; shortness of breath 59.4% vs 44.8%.

**Table** % Patients with symptomatic improvement Conventional Simulator (CS)/Virtual Simulator (VS)

| | Cough | | Sputum | | Shortness of breath | |
|---|---|---|---|---|---|---|
| | CS | VS | CS | VS | CS | VS |
| 17 | 28.6 | 42.1 | 33.3 | 43.7 | 50 | 44.4 |
| 36 | 33.3 | 35.3 | 30 | 21.4 | 62.5 | 41.7 |

The patients in the CT simulator arm tended to have a slightly worse WHO score at outset.

**Conclusion** There were differences in clinical outcome between the arm of the trial which will be discussed.

### 3.8 IMPROVED SYMPTOM RELIEF WITH FRACTIONATED PALLIATIVE RADIOTHERAPY IN PATIENTS WITH LUNG CANCER

MN Gaze, CG Kelly, GR Kerr, A Cull, RH MacDougall, GCW Howard, VJ Cowie, A Price, A Gregor, Department of Oncology, Western General Hospital, Edinburgh

**Background** Individual randomised trials have not previously suggested a benefit in survival or symptom relief from fractionated regimes of palliative thoracic radiotherapy (TRT) in patients with lung cancer.

**Aims** To determine whether fractionated TRT offers better symptom relief, quality of life or survival than single fraction TRT.

**Methods** Randomised controlled trial of 30 Gy in 10 daily fractions (F) vs 10 Gy single fraction (S) TRT. The principal endpoint was physician-assessed symptom score for cough, chest pain, dyspnoea, haemoptysis and dysphagia. Subsidiary endpoints were survival and quality of life. Symptom scores were compared using the Wilcoxon signed rank test.

**Results** 148 patients were randomised into groups matched for age, gender, histology, performance status and initial total symptom score (TSS). Patients randomised to F had lower TSS at 1st review ($P = 0.014$) and when the best TSS at either 1st or 2nd review (1 and 3 months) were compared ($P = 0.001$). This group also had better scores at either review for dyspnoea ($P = 0.010$), chest pain ($P = 0.014$) and cough ($P = 0.029$). Overall, TSS improved following TRT in 28/60 assessable patients with S and 40/57 with F ($\chi^2 = 6.64$, df = 1, $P = 0.01$). Median survival was 23 weeks with S and 28 weeks with F ($P = 0.197$). Patients with S were also more anxious than patients with F (1st review $P = 0.01$, either review $P = 0.003$).

**Conclusions** Fractionated TRT offered better symptom relief and reduced anxiety compared to single fraction palliation, but did not increase survival.

### 4.1 DNA METHYLATION IN OVARIAN CANCER

G Strathdee, K Appleton, J Plumb, R Brown, CRC Department of Medical Oncology, Beatson Laboratories, Glasgow University, Glasgow G61 1BD, UK

DNA methylation within the promoter regions of genes has been shown to be associated with transcriptional silencing. In recent years a growing list of genes have been found to be aberrantly methylated in tumours, including many genes known to be important in tumour development. We have previously shown that cisplatin resistant derivatives of the ovarian carcinoma cell line A2780 have lost MLH1 expression due to promoter methylation. Furthermore inhibition of DNA methylation results in a reduction in promoter methylation, re-expression of MLH1 and sensitisation to cisplatin and other chemotherapeutic drugs both in vitro[1] and in vivo in mouse xenografts[2]. Based on these studies, a clinical trial of the combination of carboplatin and decitabine (2′ deoxy-5-azactyidine, a DNA methyltransferase inhibitor) is scheduled to begin in early 2001.

To further define the role of aberrant methylation in ovarian cancer, we have assessed the methylation status of ten loci in a panel of 93 ovarian tumours using methylation specific PCR. Seven of the ten loci (*BRCA1*, *HIC1*, *hTR*, *MINT25*, *MINT31*, *MLH1*, *p73*) studied showed significant levels of methylation ranging between 10 and 54% of the tumours and the majority of the tumour (71%) exhibited methylation of at least one loci. Grossly normal tissue, taken from adjacent to 18 of the tumours, showed little or no evidence of methylation at the ten loci investigated. Immunohistochemical analysis of one of the genes investigated, *MLH1*, determined that methylation was associated with reduced expression in the tumours, however, immunohistochemical analysis of BRCA1 found no apparent loss of expression in tumours exhibiting methylation of this gene. Similar to reports in colon and gastric cancer, the ovarian tumours show evidence of a CpG island methylator phenotype (CIMP) in which multiple genes are concurrently methylated in a single tumour. However, unlike colon and gastric cancer, the results suggest the presence of at least two groups of $CIMP^+$ tumours, each susceptible to methylation of a different group of genes.

1. Strathdee G, MacKean M, Illand M, Brown R (1999) Oncogene 18: 2335
2. Plumb JA, Strathdee G, Sludden J, Kaye SB, Brown R (2000) Cancer Research 60: 6039
3. Strathdee G, Appleton K, Illand M, Millan DWM, Sargent J, Paul J, Brown R, American Journal of Pathology, *in press*

### 4.2 NOVEL PROTEIN INTERACTIONS OF MISMATCH REPAIR PROTEINS hMLH1 AND hMSH2

M MacPartlin[1], E. G. Homer[1], H Robinson[1], D Gillespie[2], R Brown[1], [1]CRC Department of Medical Oncology and [2]CRC Beatson Institute, CRC Beatson Laboratories, Garscube Estate, Switchback Road, Bearsden, Glasgow, G61 1BD, UK

Mismatch repair proteins have a central role in correcting mismatches in DNA occurring during DNA replication and have been implicated in engagement of apoptosis and cell cycle arrest induced by a number of important anticancer drugs. The MutS homologue, MSH2, is involved in recognising mismatches and DNA damage. The function of the MutL homologue MLH1 remains obscure, although clearly is required for mismatch repair and signalling apoptosis from DNA damage induced by agents such as cisplatin.

We have screened a yeast-two-hybrid cDNA library, from normal human breast, for proteins interacting with the MMR protein hMLH1. Amongst the interacting proteins identified was the proto-oncogene *c-MYC*. The *c-MYC* proto-oncogene and its heterodimeric partner MAX have been implicated in apoptosis, cell cycle arrest and genetic instability, although are proposed to mainly function by influencing gene transcription. The interaction between MLH1 and c-MYC is further supported by pull-down experiments using GST-hMLH1 fusion proteins and co-immunoprecipitation from human and avian cell extracts. The carboxy terminus of human and avian c-MYC, which contains the MAX binding basic region/ helix-loop-helix/"leucine zipper" (b/HLH/LZ) domain, can interact with MLH1 and a C-terminus (amino acids 515–756) fragment of MLH1. MYC mutants with C-terminal truncations of the Leucine Zipper motif of 10 and 27 amino acids, which have previously been shown to abolish MYC transforming activity and MAX binding, fail to bind MLH1.

Using a GST-Max fusion protein in pull-down experiments we have also shown that MAX is capable of interaction with the MMR protein hMSH2. The MAX:MSH2 interaction is also observed by co- immunoprecipitation of human cell extracts. Thus, it appears that c-MYC is capable of interacting with the MutL homologue MLH1, while its heterodimeric partner MAX can bind to the MutS homologue MSH2.

### 4.3 LOSS OF MGMT AND HMLH1 EXPRESSION IN SPORADIC COLORECTAL CANCER

A Jubb[1], SM Bell[1], S Gray[1], L Harkins[1], J Stahlschmidt[1], P Quirke[1], [1]Academic Unit of Pathology, Algernon Firth Building, University of Leeds, Leeds, West Yorkshire, LS2 9JT, UK

Genetic instability is at a premium during tumorigenesis, to further tumour evolution. Epigenetic inactivation of either $O^6$-methyguanine-DNA methyltransferase (*MGMT*) or the human *MutL* homologue (*hMLH1*) may result in such a mutator phenotype. *MGMT* is principally involved in the repair of alkylating lesions, which might otherwise give rise to guanine to adenine mutations, for instance in the Ki-*Ras* oncogene. *hMLH1* is involved in mis-match repair defects that are responsible for instability of homopolymeric runs on replication.

We have assessed the expression of MGMT and hMLH1 at the protein level in 284 Dukes' stage B colorectal tumours. In the analysis, we employed an automated immunohistochemistry protocol, a relatively simple technique that is applicable to large sample numbers. Total loss of MGMT staining was observed in 22% of cases. In a further 28%, positive staining in less than 50% of the tumour was recorded as 'partial loss'. Interestingly, MGMT expression was 30% lower in tumours from female cases, as compared to male cases. This is indicative of sex-specific aetiologies and molecular profiles. MGMT acts through a suicidal mechanism and, accordingly, this semi-quantitative analysis is a direct reflection on the cell's capacity for repairing alkylated DNA-adducts. hMLH1 was absent in 16% of tumours, 50% of which were negative for MGMT, suggesting these two events occur independently. Loss of hMLH1 expression was not associated with gender.

We are currently undertaking further work to determine COX-2 expression in the same series. Recent publications suggest that loss of expression from these three gene promoters may define a methylator phenotype with distinct clinico-pathological characteristics. This profile may identify patients who are less susceptible to conventional chemotherapy with 5-fluorouracil or to treatment with non-steroidal anti-inflammatory drugs. However, tumours that are negative for MGMT may be susceptible to chemotherapy with alkylating agents.

### 4.4 SUPPRESSION OF VITAMIN D SIGNALLING IN PROSTATE CANCER BY A MECHANISM INVOLVING HISTONE DEACETYLATION

KL Smith, LP O'Neill, BM Turner, MJ Campbell, Divisions of Medical Sciences, and Immunity & Infection, University of Birmingham, B15 2TH, UK

Various data support a role for 1α, 25, Dihydroxyvitamin $D_3$ (1α, 25$(OH)_2D_3$) in regulating the growth of the normal prostate gland yet prostate cancer cells appear significantly less sensitive to this action. The mechanism for this is unclear as vitamin $D_3$ receptor (VDR) content, mutational status or transcriptional activity do not correlate directly with sensitivity to 1α, 25$(OH)_2D_3$. Previously we demonstrated that inhibitors of histone deacetylation (sodium butyrate (NaB) and trichostatin A (TSA)) synergised with 1α, 25$(OH)_2D_3$ to induce apoptosis in LNCaP, PC-3 and DU-145 prostate cancer cells [1]. We hypothesized that transcriptional silencing of a subset of antiproliferative genes, by a process involving histone deactylation, altered the sensitivity to 1α, 25$(OH)_2D_3$. To elucidate further the mechanism we have treated PC-3 cells with 1α, 25$(OH)_2D_3$ (100 nM) alone or in combination with TSA (15 nM) and undertaken cDNA microarray analysis to identify changes in critical target genes and examined the acetylation of histones.

The cDNA microarray analysis revealed treatment with 1α,25$(OH)_2D_3$ alone actually upregulated many genes associated with cell cycle progression and mitosis for example, Cyclin D1 (2.4 fold), CDK4 (2.9 fold), the mitosis protein EB-1 (3.8 fold), the DNA replication factor MCMS (6.3 fold) and PCNA (4.4 fold) and the anti-apoptosis bcl-x (2.5 fold). Co-treatment with TSA down-regulated many of these events but additively or synergistically upregulated a number of growth inhibitory genes such as p21$^{(waf1/cip1)}$ (3.5 fold), the putative tumour suppressor ZO-1 (2.5 fold), and the scaffold protein zyxin (2.2 fold). Cumulatively the gene changes were consistent with the observed growth inhibition and induction of apoptosis. Furthermore, we have analysed the acetylation status of histone H4 following individual or co-treatment of agents for 2 hr. We found that 1α,25$(OH)_2D_3$ alone had no effect on the basal level of acetylation, whereas TSA showed a slight increase in the higher isoforms of acetylated H4. However, the co-treatment resulted in a dramatic and rapid increase in the levels of acetylated H4.

These data support a model whereby TSA synergises with 1α,25$(OH)_2D_3$ by hyperacetylation of histones thereby altering the transcriptional profile and restoring the normal antiproliferative signalling.

Rashid SF et al (2001). Oncogene (In press).

### 4.5 THE ROLE OF DAMAGE SENSING/PROCESSING GENES IN THE RADIATION RESPONSE OF HAEMOPOIETIC COLONY-FORMING CELLS

KP Hoyes, SA Roberts, JH Hendry, Paterson Institute for Cancer Research, Christie Hospital, Manchester M20 4BX, UK

**Purpose** Recovery of bone marrow from cytotoxic injury is mediated by the survival characteristics of its progenitor cells. The progenitor cell response is coordinated by a variety of genes in particular those involved in the regulation of DNA repair, cell cycle progression and apoptosis. We have assessed the role of a variety of relevant genes in the survival of haemopoietic progenitors after high dose-rate irradiation by comparing responses in mutant/null and wild type mice.

**Methods** Femoral bone marrow was isolated from nod/scid mice or animals null for bcl-2, bax, atm, p21 or p53 and their wild type counterparts. Marrow suspensions were irradiated in vitro with Cs-137 gamma-rays then assayed for in vitro CFC by culture in semisolid medium containing IL-3. Granulocyte/macrophage colonies were counted after 7 days growth.

**Results** CFC isolated from bcl-2 deficient mice were slightly more radiosensitive than in wild-type animals at doses to 2 Gy but not at higher doses. In contrast, CFC from bax-/-animals were more resistant than in wild-types at doses to 2 Gy but not at higher doses. CFC from nod/scid and atm-/- mice were markedly more radiosensitive, with increasing survival differentials at higher doses, whilst CFC from p53-/- animals were more radioresistant. The response of CFC from p21-/- mice was only slightly more sensitive than in wild-type animals. The results also indicate that the genetic background of the host mouse plays little role in these CFC responses, at least regarding the influence of p53 in CFC radioresponse.

**Conclusions** These data provide quantitation and comparison of the influence of these mutant and null genes, in particular those encoding (components of) the important damage- sensing gene products atm and DNA-PK, in the radiation response of haemopoietic progenitor cells. The survival-related genes bcl- 2 and bax play a role in CFC responses, which diminishes at higher doses. In contrast, the influence of atm and DNA-PK increases with increasing dose, so that these dominate the response compared to bcl-2 and bax, as well as compared to p53 and p21. The data contribute to knowledge of the molecular control of radiation response of this one normal cell type. Other cell types are also being studied.

### 4.6 INHIBITION OF MONOCYTE AND MACROPHAGE CHEMOTAXIS BY HYPOXIA AND INFLAMMATION – A POTENTIAL MECHANISM

MJ Grimsha, FR Balkwill, ICRF Translational Oncology Laboratory, St. Bartholomew's and The Royal London School of Medicine and Dentistry, Charterhouse Square, London, UK

Tumor-associated macrophages accumulate in areas of necrosis that are likely to be hypoxic. This may be because chemotaxis of monocytes and macrophages towards several chemokines is rapidly (within 60–90 min) inhibited by hypoxia. Exposure to the inflammatory cytokine TNF-α, which is also found in the tumor microenvironment, has a similar effect on macrophage migration. We report here that neither changes in mitochondrial respiration nor intracellular pH are involved in hypoxic macrophage migration arrest. However, hypoxic inhibition of migration was mimicked using chemical activators of hypoxia-inducible factor-1 and reversed by transcriptional inhibition. We used RAP-PCR, a differential display technique, to investigate which genes were upregulated within 90 minutes exposure to hypoxia. Of several thousand mRNAs screened, only one was consistently upregulated by hypoxia and this was identified as MAPK phosphatase 1 (MKP-1), which modulates MAPK activity. Levels of MKP-1 mRNA and protein were rapidly elevated in monocytic cells and primary macrophages after hypoxia or TNF-α treatment. The functional significance of MKP-1 was illustrated by hypoxia-induced decreases in phosphorylated MAPK in these cells and arrest of chemotaxis by MAPK inhibitors.

One of the important events in an 'emergency stop' response that leads to accumulation of macrophages in areas of tumor hypoxia may be inhibition of the chemoattractant signalling cascade.

### 4.7 CYTOCHROME P450 CYP1B1: A NOVEL MECHANISM OF DRUG RESISTANCE

Morag CE McFadyen[1], William T Melvin[2], Graeme I Murray[1], [1]Department of Pathology, [2]Department of Molecular and Cellular Biology, University of Aberdeen, Aberdeen, AB25 2ZD, UK

CYP1B1 is a member of the xenobiotic metabolising cytochrome P450 enzymes. This superfamily of constitutive and inducible haemoproteins are central to the oxidative metabolism of wide range of substrates including endogenous compounds involved in cell signaling, environmental carcinogens and anti-cancer drugs. Our previous studies have shown CYP1B1 protein to be to be expressed at significant levels only in tumour tissue being specifically localised to the tumour cells[1]. Our current studies are investigating CYP1B1 activity in a number of human tumours and its role in the metabolism of anti-cancer drugs in these tumours.

CYP1B1 activity can be measured by ethoxyresorufin-o-deethylase activity using the EROD assay. Our initial findings investigating ethoxyresorufin-o-deethylase activity indicate that CYP1B1 is active in a number of human tumours (100–800 fmol/min/mg of protein). Moreover, this activity can be inhibited by co-incubation with the CYP1B1 inhibitor alpha-naphthoflavone. The over-expression of active CYP1B1 in human tumours is important possibly as a mechanism of drug resistance. Several cytochrome P450 enzymes are capable of the bio-transformation of a number of anti-cancer drugs. We have recently shown several of these drugs to be substrates for CYP1B1, and our in vitro studies are now providing evidence for a functional role for CYP1B1 in drug resistance.

Using the MTT assay the cytotoxic profile of CYP1B1 with a number of anti-cancer drugs is currently being evaluated with a Chinese hamster ovary cell line known to express active CYP1B1 and a parental non-expressing CYP1B1 cell line. Our results show that on exposure to docetaxel a significant ($P$ = 0.03) increase in resistance to the cytotoxic effects of docetaxel was observed between the parental cell line ($IC_{50}$ = 22 nM) and the cell line expressing CYP1B1 ($IC_{50}$ = 100 nM). In addition, co-incubation with alpha-naphthoflavone, reversed the resistance to docetaxel in the CYP1B1 expressing cells. The resistance to the cytotoxic effects of docetaxel in those tumours expressing CYP1B1 may have important clinical implications. It is also likely that the over-expression of active CYP1B1 is a general mechanism of drug resistance. Moreover, the ability to overcome this drug resistance with the appropriate CYP1B1 inhibitors could be developed clinically.

1. Murray GI, Melvin WT, Greenlee WF, Burke MD. 2001 *Annu Rev Pharmacol Toxicol* **41** (in press).

### 4.8 TUMOUR PHYSIOLOGY AS AN IMPORTANT AND NEGLECTED CAUSE OF DRUG RESISTANCE

IF Tannock, Princess Margaret Hospital and University of Toronto, Toronto ON M5G 2M9, Canada

Clinical drug resistance of solid tumours may be caused by cellular mechanisms (e.g. P-glycoprotein, Pgp); by mechanisms that depend on the microenvironment, especially the requirement for drugs to penetrate tumour tissue to reach all of the target cells; and on repopulation of tumour cells between courses of chemotherapy.

We have grown tumour cells on collagen-coated porous teflon membranes to form multicellular layers (MCL) up to 200 μm thick. MCL share features of solid tumours including rare tight junctions and an extracellular matrix. We have studied the time-dependent penetration through MCL of several anticancer drugs used commonly for the treatment of solid tumours: cisplatin, doxorubicin, 5FU, gemcitabine, methotrexate, mitoxantrone, paclitaxel and vinblastine. Penetration of all drugs is slow compared with that through the teflon membrane alone and is particularly poor for the weak bases doxorubicin and mitoxantrone (< 10% penetration compared to the teflon membrane alone at 6 hours). Factors that limit net uptake of drug into cells (including expression of Pgp) increase drug penetration through tissue, while reversal of Pgp decreases tissue penetration of its substrates. The tissue penetration of doxorubicin and mitoxantrone may be improved substantially by agents that inhibit the sequestration of these drugs in acidic endosomes of cells.

Repopulation of surviving tumour cells between successive treatments is a process known to influence outcome of radiotherapy and is likely to be even more important in the longer intervals between cycles of chemotherapy. It might be inhibited selectively by biological agents such as inhibitors of growth factor receptors that are expressed on tumour cells.

The literature on drug resistance is dominated by studies of molecular mechanisms. While important, other causes of effective drug resistance such as limited tissue penetration by drugs and repopulation of tumour cells between cycles of chemotherapy may be (a) equally important and (b) susceptible to modification to improve therapeutic index.

Supported by grants from the Canadian Institute of Health Research and the National Cancer Institute of Canada.

### 5.1 THE WNT-APC-β CATENIN PATHWAY IN PHYLLODES TUMOURS

EJ Sawyer[1,2,3], AM Hanby[2], C Gillett[2], A Rowan[1], R Poulsom[4], P Ellis[3], IPM Tomlinson[1], [1]Molecular and Population Genetics Lab & [4]Histopathology Unit, ICRF, 44 Lincoln's Inn Fields, London WC2A 3PX, [2]Hedley Atkins/ICRF Breast Pathology Laboratory, [3]Guys, Kings, St Thomas[1] Cancer Centre, Guy's Hospital, London SE1 9RT, UK

Phyllodes tumours are uncommon fibroepithelial mammary lesions which may show a spectrum of behaviour from benign through to sarcomatous transformation. In a previous study of phyllodes tumours we have shown that both the stroma and epithelium contain distinct molecular changes suggesting that both are part of the neoplastic process. In view of this finding we decided to study stromal-epithelial interactions in these tumours by examining the Wnt-APC-β catenin pathway.

β catenin and cyclin D1 immunohistochemistry was performed on 119 tumours. 72% of tumours had nuclear β catenin staining in the stroma and in 57% this was moderate or strong. In 17 cases (14%) this nuclear staining was more prominent in the stromal cells adjacent to the epithelium. Of the 8 malignant tumours in the series, 7 showed no or weak nuclear staining and this relationship was statistically significant ($P < 0.025$). 19% of the phyllodes tumours showed moderate or strong cyclin D1 staining and this correlated with nuclear stromal β catenin staining ($P = 0.05$).

46 of the tumours were analysed for β catenin mutations using SSCP and sequencing. No β catenin mutations were found in any of the tumours. Loss of heterozygosity (LOH) of the microsatellite marker, D5S346, was used to infer APC mutation. Only one phyllodes tumour showed LOH at D5S346 and this tumour had moderate nuclear β catenin staining.

Wnt 5a expression was detected using in situ hybridisation. Thirteen cases were chosen to reflect the different β catenin staining patterns (10 benign, 3 malignant). All tumours showing strong nuclear β catenin staining expressed epithelial Wnt 5a ($P < 0.0015$) and in the 2 cases with the subepithelial distribution of β catenin staining there were also subepithelial clusters of Wnt 5a expression.

This study shows that abnormal nuclear β catenin accumulation is common in the stroma of benign phyllodes tumours and that one of the down stream targets of this is cyclin D1. Significantly in malignant tumours this abnormal β catenin expression is lost. The correlation of strong β catenin staining and Wnt 5a overexpression together with the similar subepithelial distributions suggests that epithelial Wnt 5a signalling drives proliferation of the stroma in benign phyllodes tumours and that proliferation becomes Wnt independent in malignant tumours.

### 5.2 ADRIAMYCIN/CMF (A/CMF) FOR HIGH RISK BREAST CANCER IN THE UK IS AS GOOD AS (CALGB9344) AC/TAXOL)-ANGLO-CELTIC COOPERATIVE ONCOLOGY GROUP (ACCOG)

RCF Leonard[10], E Toy[1], T Evans[2], J Levay[3], I Kennedy[4], R Grieve[5], T Perren[6], A Jones[7], J Mansi[8], J Crown[9], G McIntyre[10], A Anderson[10], S Povey[10] and D Cameron[10], Edinburgh[10], Cardiff[1], Glasgow[2], Ipswich[3], Hamilton NZ[4], Coventry[5], Leeds[6], London[7,8], Dublin[9]

The Milan A/CMF 12 cycle regimen remains the most effective adjuvant treatment policy with published long-term disease-free and overall survival data for multiple node-positive breast cancer. It was adopted by ACCOG as the standard arm of its first high dose trial in high risk disease (ACCOG1). The data from this completed trial are not yet mature but the regimen has been used in many of our centres as a standard protocol for high risk disease outside the trial. From 6 UK Irish and New Zealand centres, 328 patients with stage 2 disease received 9–12 courses of A/CMF combined in 287 cases with adjuvant radiotherapy, routinely between courses 5 and 6. Median dose delivered was 100% of planned but in 40 patients there were 1–10 weeks aggregate dose delays and in 171 patients, dose reductions. Main toxicities were alopecia (which typically recovered during CMF phase) and grade 3 or 4 neutropenia which in 19 patients caused sepsis. There were no treatment-induced deaths.

This was a particularly poor risk population; only 2 were node–ve, 16 were 1–3 node+ve; 178 4–9 node+ve and 150 10+ node+ve. 95 were ER–ve and 15 of these and all ER +ve had tamoxifen for 5 years on completion of chemotherapy.

At a median follow-up of 2.8 years, there have been 19 local relapses, 87 distant relapse and one patient with both. Relapse free and overall survival are shown with the CALGB 9344 data shown for comparison.

| Regimen | Node number | 2 YR DFS | 2 YR OS |
|---|---|---|---|
| AC/TAX | 4–9 | 85% | 94% |
| A/CMF | 4–9 | 85% | 93% |
| AC/TAX | 10+ | 69% | 85% |
| A/CMF | 10+ | 75% | 90% |

This audit is further evidence of the efficacy, tolerability and safety of a regimen that should be a gold standard against which any complex and expensive regimen requires to be tested; for example the recently launched 'TACT' trial.

### 5.3 ADJUVANT TAXANES FOR EARLY BREAST CANCER – CLINICAL UNCERTAINTY EXISTS

P Barrett-Lee[2], J Bliss[1], P Ellis[3], E Hall[1], L Johnson[1], D Lawrence[1], [1]CTSU, Section of Epidemiology, Institute of Cancer Research, Sutton, [2]Velindre Hospital, Cardiff, [3]Guy's, King's & St Thomas's Hospital, London, on behalf of the TACT Trial Management Group[†]

The Taxotere as Adjuvant Chemotherapy (TACT) Trial tests whether there is an increase in 5-year disease-free and overall survival of early breast cancer patients randomised to receive 4 cycles of sequential docetaxel (Taxotere) following 4 cycles of FEC, compared with UK anthracycline-based chemotherapy of similar duration. In November 2000, a questionnaire circulated to 205 potential TACT Trial participants based at 94 UK centres aimed to a) confirm that target recruitment is achievable, b) ascertain whether a choice of control arm ($8 \times$ FEC or E-CMF) is necessary, and c) establish the level of clinical uncertainty about the use of adjuvant taxanes for different patient groups.

**Target recruitment** 132 replies from clinicians at 86 centres were received (114 within 2 weeks). The combined anticipated annual accrual was 2431. Even if this level were halved, target recruitment (3340 over 3 years) is achievable.

**Choice of control arm** 83 (62%) clinicians preferred FEC as the control arm and 49 (38%) E-CMF. This translates to 1436 and 995 patients per year respectively.

Of 123 respondents who indicated whether the withdrawal of their chosen control arm would affect their recruitment, 60 (49%) of respondents (contributing 1269 (54%) patients) suggested that it would; 63 (51%) of respondents (contributing 1095 (46%) patients) suggested it would not. Withdrawal of the FEC control arm would result in the loss of up to 767 (33%) potential patients. Withdrawal of the E-CMF control arm would result in the loss of up to 540 (23%) potential patients.

**Level of clinical uncertainty for taxane usage** Uncertainty was greatest in the <50 age group, and for node +ve patients aged 50–70. For patients aged <50, ER –ve, with 1–3 nodes +ve, 88% of respondents expressed clinical uncertainty. Only 1% of respondents expressed clinical uncertainty for patients aged >70, ER +ve, and node –ve.

**Conclusions** Recruitment into the TACT Trial should meet or exceed target. Clinical opinion is divided over the choice of control arm, however more respondents opted for $8 \times$ FEC than E-CMF. Clinical uncertainty about the use of adjuvant taxanes is widespread and varies greatly according to patient characteristics.

[†]D Bloomfield, L Branston, M Brunt, D Cameron, D Dodwell, H Earl, L Foster, A Harnett, J Hearn, P Hopwood, J Houghton, S Houston, A Jones, S Johnston T Perren, C Poole, I Smith, M Stead, C Twelves, M Verrill, J Yarnold

### 5.4 CAPECITABINE NAMED PATIENT PROGRAM FOR PATIENTS WITH ADVANCED BREAST CANCER: THE UK EXPERIENCE

RCF Leonard, A Anderson[1], R Salazar, C Twelves[2], A Hutcheon, D Bissett[3], T Bates[4], A Chaturvedi[5], S Chan, J Carmichael[6], on behalf of the UK Capecitabine Audit Group, [1]Edinburgh, [2]Glasgow, [3]Aberdeen, [4]Oxford, [5]Hull, [6]Nottingham, UK

102 patients with advanced breast cancer received capecitabine in a UK open access programme and have been analysed for response and toxicity. Median age was 53.2 (range 30–95). Patients had received between 0–4 prior chemotherapy regimens for advanced disease. 58% of patients had visceral disease and median number of sites of disease was 1. 60.8% had previously received anthracyclines, 25.5% taxoids and 6.9% infusional 5-FU. A median of 5 cycles were given.

Dose reductions occurred in 32.4% of patients (10.2% of cycles). The mean dose intensity was 95%. There were 3 complete responders, 17 partial responders, and the total objective response rate was 19%. Stable disease was achieved in 46% and progression was seen in 30%. Toxicity is tabulated.

| Event | Grade 1 (%) | Grade 2 (%) | Grade 3 (%) | Grade 4 (%) |
|---|---|---|---|---|
| Neutropenia | 2.0 | 1.0 | 2.0 | 1.0 |
| Thrombocytopenia | 3.9 | 2.0 | 1.0 | 0.0 |
| Mucositis | 1.0 | 2.9 | 2.0 | 0.0 |
| Fatigue | 12.7 | 3.9 | 2.9 | 1.0 |
| PPE | 15.7 | 11.8 | 7.8 | 0.0 |
| Diarrhea | 21.6 | 5.9 | 4.9 | 2.0 |
| Nausea | 23.5 | 5.9 | 1.0 | 0.0 |
| Vomiting | 11.8 | 2.9 | 2.0 | 0.0 |

We conclude that capecitabine was well tolerated and active in extensively pretreated patients with advanced breast cancer. Toxicity was manageable at the recommended dose of 1250 mg/m$^2$ b.i.d. for 14 days q21 days.

### 5.5 INFUSIONAL 5-FLUOROURACIL AND VINORELBINE FOR METASTATIC BREAST CANCER – A NEW REGIMEN WITH HIGH ACTIVITY

N Stuart[1], M McIllmurray[2], SO'Reilly[3], C Price[4], S Johnston[5], J Joffe[6], F Neave[7], T Perren[8], T Crosby[9], [1]Ysbyty Gwynedd Bangor, [2]Lancaster Royal Infirmary, [3]Clatterbridge Centre for Oncology, [4]Bristol Centre for Oncology, [5]Royal Marsden Hospital, [6]Huddersfield Royal Infirmary, [7]North Middlesex Hospital, [8]St James' Hospital, Leeds, [9]Velindre Hospital, Cardiff, UK

An increasing number of drugs are available for the treatment of metastatic breast cancer (MBC) but the optimum regimen is not defined. There remains a need for regimens that are active and well tolerated particularly after anthracyclines (A) or taxanes. Vinorelbine (VRB) has shown high single agent activity and low toxicity. 5-fluorouracil (5FU) given by continuous infusion (CI) has shown activity in heavily pretreated MBC, and has a different toxicity profile to VRB. Sixty-four patients (pts) with MBC have been enrolled in a phase II study of VRB 30 mg/m$^2$ day 1 and 8 each 21 days and 5FU 200 mg/m$^2$/day given by CI. Data is available on the first 31 pts. All pts had received prior treatment with A (27) or mitoxantrone (4), 4 as adjuvant therapy with relapse within 12 months and 27 for metastatic disease. 10 pts had 2 or more prior regimens. All pts had measurable disease, ECOG PS $\leq$ 2, and normal liver and renal function. Median age was 51 (27–78). Achieved overall dose intensity was VRB 15 mg/m$^2$/week (75%), 5-FU 157 mg/m$^2$/day (78%) with delay and dose reduction largely due to neutropenia. 11 episodes of neutropenic sepsis occurred but no treatment related deaths. 10 patients suffered significant problems from their subclavian lines (thrombosis 3, displacement 3, pneumothorax 1, infection 1, other 2) and 5 had to stop treatment because of this. Incidence of other toxicity was generally low. Grade 1–2 and 3–4 toxicity occurred with the following incidence. Neutropenia 19%, 65%, thrombocytopenia 29%, 0%; anaemia 94%, 0%, nausea and vomiting 23%, 7%; mucositis 23%, 4%; constipation 19%, 4%; peripheral neuropathy 17%, 3%; diarrhoea 14%, 4%; hand foot syndrome 7%, 5%. Tumour response occurred in 17 (54%) of patients. Median duration of response in responding pts was 18 weeks. VRB + 5FU by CI is active and generally well tolerated in MBC after treatment with anthracyclines. Neutropenia is the main toxicity. Future studies will explore the role of oral vinorelbine and the oral 5FU analogues with the aim of developing an active, well tolerated and convenient oral regimen for MBC.

### 5.6 IMPACT OF YOUNG AGE ON LOCAL RECURRENCE AFTER BREAST CONSERVING THERAPY

IH Kunkler[1], W Jack[1], G Kerr[1], U Chetty[2], M Dixon[2], T Anderson[2], A Rodger[3], RCF Leonard[1], APM Forrest[2], [1]Dept of Clinical Oncology, [2]Edinburgh Breast Unit, Western General Hospital, Edinburgh, [3]Monash University, Victoria, Australia

**Background** There is increasing evidence that young age may be an independent risk for local recurrence (LR) after breast conserving surgery (BCS) and postoperative breast irradiation (RT).

**Aim** A retrospective audit of BCS + RT was undertaken to assess the impact of prognostic factors including age on the risk of LR.

**Methods** Between 1981–1993, 929 patients with T1-2, NO-1, MO breast cancer were treated at the Western General Hospital by BCS and RT. 729 [79.2%] were carcinomas of no special type, 45 [6.9%] lobular and 58 [6.3%] tubular. Mean tumour size was 23.7 mm (range 3–50 mm). Median age was 53 years. Minimum follow up was 6 years, apart from 2 patients lost to follow up at 3 years. Re-excision of the margins was carried out in 118 patients. Axillary surgery was either by a level 3 clearance (166 [18%]) or a 4-node lower axillary node sample (721 [78.3%]) followed by axillary RT in N+ [28%]. Standard radiotherapy was 45 Gy in 20 fractions over 4 weeks with a boost (864) by electrons (595 [60.9%]) or iridium implant (252 [29.2%]). Axillary RT was given to 472 patients. 609 [66%] were treated with adjuvant tamoxifen and 79 [8.6%] with CMF. Local failure was defined as relapse in the breast. Distant failure included patients with concurrent local relapse.

**Results** A local relapse occurred in 82 patients, distant relapse in 178 patients and regional relapse in 78 patients. The 5 year actuarial breast relapse rate was 4.8% at 5 years, 10.1% at 10 years and 14.6% at 15 years. The 5 year local relapse by age cohort is shown in the Table.

**Table:** Breast relapse rate by age cohort

| < 30 years | 30–39 years | 40 –49 years | 50–59 years | 60–69 years | 70+ years |
|---|---|---|---|---|---|
| 33.3% | 14% | 5.7% | 2.6% | 1.8% | 12.2% |

**Conclusion** Breast relapse rates in women under the age of 40 were particularly high, even with attention to obtaining clear margins. These higher rates may reflect in part undetected multifocality in the radiodense breast in younger women. The higher risks of breast relapse in young women should be explained to patients in whom breast conserving therapy is being considered.

### 5.7 USING HISTOPATHOLOGIC DATA TO IMPROVE THE DETECTION RATE OF GERMLINE *BRCA1* MUTATIONS

G Mitchell[1,2], D Easton[3], R Pears[4], J Harroway[5], RA Eeles[1,2], [1]Institute of Cancer Research, Surrey, UK, [2]Royal Marsden NHS Trust, London, UK, [3]CRC Strangeways Research Laboratories, Cambridge UK, [4]St George's Hospital, London UK, [5]Christchurch Hospital, Christchurch, New Zealand

**Aim** To improve the selection of families for diagnostic genetic testing for germline mutations in the *BRCA1* gene by using pathologic data from breast cancer histopathology reports to enrich risk estimates based on pedigree data.

**Introduction** Most UK cancer genetics clinics offer *BRCA 1/2* mutation testing to families with $\geq$ 4 cases of young onset breast and/or ovarian cancer (*a priori* probability > 60% using Claus risk estimates) to ensure a cost-effective mutation detection rate. A family has to be of sufficient size to fulfil these stringent criteria, potentially excluding an important number of families with mutations. Incorporating pathologic features of breast cancer may help to improve prediction of mutation status as pathologic differences have been detected between sporadic and *BRCA1*-associated breast cancers.

**Methods** 226 individuals undertaking a diagnostic test for germline *BRCA1* mutations between June 1991 and March 1999 at the Royal Marsden, St George's and Guy's Hospitals, and for whom histopathology records could be identified, were studied.

**Results** 20 germline *BRCA 1* mutations were detected. Mutation carriers were of significantly higher tumour grade than non-carriers. Vascular invasion, histological phenotype and DCIS were not predictive of mutation status. Table 1 summarizes the distribution of *BRCA 1* mutations using Claus risk estimates, presence of ovarian cancer and pathologic grade. 30% of mutation carriers were 'low risk' but all had grade 3 tumours. Combining tumour grade with family history discriminated well between 132 lower risk families, potentially excluding 34% of them ('low risk', pathological grades 1&2) from genetic testing.

**Conclusions** The addition of pathological grade helps identify families with *BRCA 1* mutations which do not fulfil UK pedigree criteria for mutation testing with potential personal benefit to these families and cost benefit to the NHS

**Table 1**

| | No. patients *BRCA 1* mut+ (% of all *BRCA1* carriers) | No. of patients *BRCA1* mut– (% of all non-*BRCA1* patients) |
|---|---|---|
| Claus risk >90% ovarian cancer+ | 5 (25) | 12 (6) |
| Claus risk >90% ovarian cancer- | 5 (25) | 49 (24) |
| Claus risk <90% ovarian cancer+ | 4 (20) | 19 (9) |
| Claus risk <90% ovarian cancer- | 6 (30) | 126 (61) |
| *Tumour: grade 1* | *0 (0)* | *10 (5)* |
| *grade 2* | *0 (0)* | *33 (16)* |
| *grade 3* | *6 (30)* | *59 (29)* |
| *Unknown grade* | *0 (0)* | *10 (5)* |

### 6.1 THE TERMINAL 11A.A. CYTOPLASMIC TAIL OF THE β6 INTEGRIN SUBUNIT IS ESSENTIAL FOR THE PROMOTION OF αvβ6-DEPENDENT INVASION

MR Morgan*, GJ Thomas[1], PM Speight[1], IR Hart, JF Marshall, Richard Dimbleby Department of Cancer Research/ICRF Lab, St. Thomas' Hospital, London, Eastman Dental Institute, University of London, UK

The integrin αvβ6 is expressed de novo on keratinocytes in both squamous cell carcinoma (SCC) and wound healing, which are both entities involving invasive behaviour. Previously we have shown that upregulation of αvβ6, the fibronectin/tenascin receptor, promotes a more malignant phenotype in SCC cells involving increased invasive and migratory capabilities.

The β6 subunit has an unique C-terminal 11 amino acid sequence which may be responsible, through intracellular signalling, for these altered functional effects in SCC cell lines expressing high levels of αvβ6.

To investigate this possibility we have created a panel of SCC cell lines, using retroviral transfer of appropriate cDNAs, which express either the wild-type β6 or a mutant β6 lacking just the terminal 11 a.a. tail. VB6 and V3β6Δ11 aa cell lines were generated which express similar levels of αvβ6; a cell surface level approximately 10 times as that expressed by the null transfectant cell line, C1.

Haptotactic migration assays, toward fibronectin, showed similar levels of migration (11.53% + 11.78%) by both the VB6 and the V3β6Δ11aa cell lines. The migration of V3β6Δ11aa was reduced to C1 levels by the αvβ6 blocking antibody, 10D5, demonstrating that the truncated β6 subunit in the V3β6Δ11aa cell line still functions as a fibronectin receptor.

In marked contrast, however, in transwell invasion assays VB6 cells invaded through Matrigel to a significantly greater extent (100%) than the C1 null transfectant cell line (33.9%) and the V3β6Δ11aa line (36.2%). This invasive behaviour was αvβ6 and MMP-9 dependent and suggests that the unique 11 a.a. extension of β6 is essential for this more aggressive phenotype. Densitometric analysis of zymography results showed that C1 and V3β6Δ11aa cells exhibited reduced levels (46.4% + 49.4% reductions) of secreted MMP-9 activity when compared with VB6 cells. Thus, the terminal 11 a.a. sequence of β6 integrin promotes invasion, at least in part, by increasing the expression of activated MMP-9.

### 6.2 ASSOCIATION OF SCAR WITH THE SRC TYROSINE KINASE: CONSEQUENCES FOR SCAR REGULATION

VG Brunton[1], P Timpson[1], NO Carragher[1], L Machesky[2] and MC Frame[1], [1]Beatson Institute for Cancer Research, Switchback Road, Glasgow, G61 1BD, [2]School of Biosciences, University of Birmingham, Birmingham, B15 2TT, UK

The Arp2/3 complex regulates the assembly of new actin filament networks at the leading edge of cells where it is located in a variety of cell types. Proteins of the WASP family and related protein group the Scars, bind directly to the Arp2/3 complex and stimulate its ability to promote the nucleation of new actin filaments in response to Cdc42 and Rac activation. These proteins are thought to act as intermediates between growth factor receptors, via adaptor molecules such as Nck and Grb2, and the actin cytoskeleton, however their regulation within the cell is not fully understood. The non-receptor tyrosine kinase Src phosphorylates and associates with a number of proteins involved in the regulation of the actin cytoskeleton and the aim of this study was to establish whether Src regulates the activity of Scar1. Using a GFP-tagged Src we have shown that the localisation of Src within the cell is under the control of the Rho GTPases: Src localises to focal adhesions in a Rho-dependent manner and subsequent activation of Rac or Cdc42 results in its localisation to lamellipodia or filopodia respectively. Using immunofluorescence we have also demonstrated that Scar colocalises with Src at focal adhesions, lamellipodia and filopodia. Scar associates with Src in immune complexes, which is mediated via the SH3 domain of Src. The association of Scar with Src results in the phosphorylation of Scar and we are currently assessing whether this alters the ability of Scar to associate with the Arp2/3 complex. We have also found that Scar is cleaved by calpain, a protease found in focal adhesions, whose activity is regulated by Src (Carragher et al, J. Biol. Chem. 276: 4270–4275, 2001). This represents another mechanism whereby Scar activity may be regulated within the cell.

### 6.3 EXPRESSION OF MAP KINASE KINASE-5 (MEK5) IN HUMAN PROSTATE CANCER

BL Jenkins, PB Mehta, CN Robson, DE Neal, HY Leung, Department of Surgical and Reproductive Sciences, Medical School, University of Newcastle-Upon-Tyne, NE2 4HH

MAP kinase kinase-5 (MEK5) is a protein kinase upstream of the MAP kinases. It has a pivotal role in cell signalling[1]. MEK5 specifically phosphorylates ERK5 which activates the MEF2 transcription factors. A consequence of this is increased transcription of *c-jun* and cellular proliferation[2,3]. Previous work by our group has demonstrated the loss of a DNA fragment in the highly metastatic cell line LNCap-LN3 when compared to the parental cells. The fragment showed 100% homology to the 3'untranslated region of *mek5*. Immunostaining for MEK5 has been performed on a range of resected prostate cancer samples to further delineate its function.

**Materials and Methods** Western blotting was performed on lysates of the above cell lines to identify MEK5 protein expression. The filter was probed with three anti-MEK5 antibodies to select the most efficacious one. 128 resected human prostate cancer specimens and 10 benign hypertrophy specimens were then immunostained. The intensity of staining was graded with two independent observers as negative/weak or moderate/strong and the results compared with Gleason grade, presence of metastases and survival.

**Results** The BPH specimens all stained very negative/weak for MEK5. Of the cancer specimens with Gleason grade of 4–7, 10 specimens (29.4%) stained negative/weak and 24 (70.6%) moderate/strong. In the Gleason grade 8–10 group, 14 (14.9%) stained negative/weak and 80 (85.1%) moderate/strong. Fishers exact test revealed that this staining was significantly different ($P$ = 0.05). A Kaplain-Meier Survival plot revealed that higher MEK5 staining was associated with lower survival than the group with less MEK5 staining, though not significantly different ($P$ = 0.625). Higher MEK5 staining intensity was seen in patients with metastases compared to those without ($P$ = 0.03). Survival of patients with metastases was significantly less than those without ($P < 0.0001$). A survival plot with the two Gleason grade groups, Gleason 2–7 and 8–10 and revealed survival was significantly different ($P$ = 0.008).

**Discussion** MEK5 acts within a very specific cell signalling protein kinase cascade. The loss of the *mek5* fragment in the LNCaP-LN3 line may suggest a role for this gene in metastasis. MEK5 protein expression is low in BPH, and up-regulated in prostate cancers. Intense staining is seen in specimens with high Gleason grades and in patients with metastases. High MEK5 expression is associated with poor survival.

1. Zhou G, Bao ZQ, Dixon JE *J Biol Chem* **270(21)**: 12665
2. Kato Y, Kravchenko VV, Tapping RI et al. *EMBO J*., **16(23)**: 7054
3. Katto Y, Tapping RI, Huang S et al. *Nature* **395(6703)**: 713

### 6.4 FGF-2 MEDIATES SURVIVAL OF SMALL CELL LUNG CANCER CELLS (SCLC) THROUGH A MEK-DEPENDENT PATHWAY: CORRELATION WITH THE TRANSLATIONAL REGULATION OF BCL-2 FAMILY MEMBERS

OE Pardo[1], G Salerno[1], S Raguz[2], MJ Seckl[1], [1]Cancer Cell Biology Unit, [2]MRC Lab., Hammersmith Hospital, London W12 0NN, UK

The role of basic fibroblast growth factor (FGF-2) has not previously been investigated in the biology of small cell lung cancer (SCLC), a disease with a 5-year survival < 5%. Here we show that FGF-2 protects SCLC cells from drug-induced cell death. Etoposide [0.1 µM], one of the main drugs used in SCLC treatment, induced apoptosis in H510 SCLC cells as demonstrated by annexin V staining, cytochrome c release, caspase-3 activation and PARP cleavage. Addition of FGF-2 [0.1 ng/ml], 4h prior to etoposide, inhibited the appearance of these markers and rescued the cells from apoptosis. As FGF-2-induced the activation of both the $p42/44^{MAPK}$ and the $p70^{S6K}$, we tested whether these signalling pathways could be involved in preventing etoposide-induced apoptosis. Inhibition of the $p42/44^{MAPK}$, using a MEK inhibitor (PD098059) abolished the prosurvival properties of FGF-2. However, inhibition of $p70^{S6K}$ or the PI3K/PKB pathway, with rapamycin or Ly294002 respectively, did not block FGF-2 induced survival. Time course experiments revealed that a minimum of 3h incubation with FGF-2, prior to etoposide treatment, was needed to rescue cells from apoptosis. This did not correlate with the transient activation of $p42/44^{MAPK}$ (peak at 5 min and back to control levels by 10 min) suggesting that induction of new protein synthesis was required. This was confirmed by the observation that cycloheximide, a protein synthesis inhibitor, abolished the prosurvival effect of FGF-2. We further demonstrate that the anti-apoptotic proteins Bcl-2 and Bcl-$X_L$ was neither due to protein stabilisation (as assessed by pulse chase) nor to an increase in mRNA synthesis (as assessed by real time quantitative PCR), suggesting that FGF-2 regulates the levels of these proteins at the translational level. This was further confirmed by the lack of effect of actinomycin D on upregulation of these proteins. In addition to its effect on the anti-apoptotic members of the Bcl-2 family, FGF-2 was able to inhibit the induction of the proapoptotic member, Bad, by etoposide. Thus, FGF-2 appears to act on both sides of the survival/death balance, tilting it towards cell survival. These data suggest that FGF-2 could be involved in SCLC cell resistance to chemotherapy and inhibition of FGF-2 signalling may provide new ways of sensitising cells to treatment.

### 6.5 CHRONIC HYPOXIA RESULTS IN DOWNREGULATION OF PRO-APOPTOTIC PROTEINS OF THE BCL-2 FAMILY IN A PANEL OF HUMAN COLON CARCINOMA CELLS

J Erler[1], K Williams[2], I Stratford[2], C Dive[1], [1]CRC Molecular Pharmacology Group, School of Biological Sciences, [2]Experimental Oncology Group, School of Pharmacy and Pharmaceutical Sciences, University of Manchester, Oxford Road, Manchester M13 9PL, UK

Hypoxic tumours are more likely to have a poor prognosis due to resistance to radio- and chemotherapy. These hypoxic tumours are also associated with a higher degree of malignancy and increased ability to metastasise. Cancer cells sense hypoxia and respond by altering the expression of a variety of genes, primarily through a HIF-1 dependent trans-activation of hypoxia responsive elements (HREs). Some of these hypoxia-regulated genes are important for tumour growth and include VEGF, glucose transporters, glycolytic enzymes and enzymes important in maintaining cellular pH homeostasis. There is overwhelming evidence to show that HIF-1 deficiency adversely effects tumour growth (e.g. Maxwell et al (1997) PNAS, 94: 8104–8109). Since tumour growth is a balance between tumour cell proliferation and cell loss (cell death), we have sought to define the contribution of hypoxia/HIF-1 dependent processes that are important in apoptosis. It is known that the Bcl-2 family of proteins play a central role in regulating the threshold for drug-induced apoptosis. We therefore examined whether chronic hypoxia would modulate the expression level of Bcl-2 homologs in a panel of colon carcinoma cell lines (HCT116, SW480 and HT29). These cell lines each generated elevated levels of VEGF protein under hypoxic conditions and these preliminary observations would suggest the colon lines are HIF-1 proficient. Moreover, levels of five of the pro-apoptotic family proteins Bid (full length and truncated), Bad, Bax, Bak and Nip3 were decreased in all three cell lines exposed to hypoxia for 16 h albeit to different extents. No effect on the expression of the anti-apoptotic proteins Bcl-2 and Bcl-xL was detected. The level of expression of these pro-apoptotic genes in tumours in vivo is currently being assessed together with their spacial location in relation to expression of intrinsic markers for hypoxia such as GLUT-I and CA IX.

### 6.6 FACTORS REGULATING THE PHOSPHORYLATION OF PROTEIN KINASE B/AKT IN HUMAN PROSTATIC CELL LINES

R Michael Sharrard, Norman J Maitland, YCR Cancer Research Unit, Department of Biology, University of York, York YO10 5YW, UK

Protein Kinase B (PKB), also known as Akt, is a key enzyme regulating cell survival, motility, and gene transcription. Its downstream effectors include the apoptotic regulator protein BAD, the Forkhead transcriptional regulators, GSK-3, and caspase 9. PKB/Akt is itself regulated by phosphorylation at serine 473 and threonine 308; the phosphorylation of these residues may be regulated by separate pathways and may have differential effects on pathways downstream of the enzyme. PKB/Akt phosphorylation is influenced by soluble growth factors and by integrin-based cell-substrate adhesion via signalling pathways involving ras, integrin-linked kinase (ILK), and phosphatidylinositol 3-kinase (PI3K) and its product PIP3. PTEN tumour-suppressor protein, which dephosphorylates PIP3 as well as specific protein targets, may exert many of its effects through regulation of PKB/Akt.

We studied the effects of integrin-based cell-substrate adhesion and serum-derived growth factors on the phosphorylation status of PKB/Akt in cell lines derived from human non-tumour prostate epithelium (PNT2, PNT1a), from a well-differentiated prostate tumour (P4E6), and from prostate tumour metastases (LNCaP, PC3). Phosphorylation of PKB/Akt was assayed by Western blotting and probing with antibodies specific to the ser473-and thr308-phosphorylated protein forms. In the non-tumour cells, maintenance of ser473 phosphorylation was dependent upon both adhesion to a solid substrate and the presence of serum, while the tumour cells showed only small decreases in ser473 phosphorylation in response to prevention of adhesion or serum deprivation. In contrast, both advanced tumour cell lines were extremely sensitive to the PI3K inhibitor LY294002 (ser473 phosphorylation reduced by 94% in LNCaP and by 86% in PC3), while PNT2, PNT1a and P4E6 cells were markedly less sensitive. Phosphorylation of thr308 was almost undetectable in PKB/Akt from all the cell lines except LNCaP, in which thr308 phosphorylation showed the same pattern of susceptibility as ser473.

These results suggest that the regulation of PKB/Akt in normal prostatic epithelial cells is effected through a convergence of signalling pathways from adhesion proteins and receptors for soluble growth factors. PKB/Akt regulation in tumour cells is relatively independent of these extracellular signals. The sensitivity of LNCaP and PC3 cells to the PI3K inhibitor suggests a mechanism in which PI3K activation has become uncoupled from cell surface receptor signalling and is the predominant factor in maintaining PKB/Akt phosphorylation. These studies suggest that drugs which inhibit PI3K and its products may be selectively therapeutic in treatment of advanced prostate cancer.

### 6.7 MOLECULAR ANALYSIS OF BAG-1 FUNCTION IN HUMAN BREAST CANCER CELLS

PA Townsend, R Cutress, M Brimmell and G Packham, CRC Wessex Medical Oncology Unit, Cancer Sciences Division, School of Medicine, University of Southampton, Southampton SO16 6YD, UK

BAG-1 is a multifunctional protein, which associates with a variety of cellular targets including steroid hormone receptors, BCL-2, RAF-1 kinase, the hepatocyte growth factor receptor, the proteasome and 70 kDa heat shock proteins, HSC70 and HSP70. BAG-1 promotes cell survival, proliferation and metastasis, alters responses to steroid hormones and modulates the chaperone function of HSC/HSP70. BAG-1 exists as three distinct isoforms, BAG-1/S, BAG-1/M and BAG-1/L, which originate from a single mRNA by alternate translation initiation and are differentially localised in cells. Our immunohistochemical analyses have demonstrated that BAG-1 is overexpressed in the majority of invasive breast carcinomas. Nuclear, but not cytoplasmic expression of BAG-1 is significantly inversely correlated with tumour grade and tends to associate with improved overall survival. We have generated expression constructs that selectively encode individual BAG-1 isoforms and in long-term clonogenic assays, overexpression of each of the BAG-1 isoforms protects MCF7 cells from the growth inhibitory effects of heat shock (HS). BAG-1S also protects against growth inhibition by some but not all chemotherapeutic drugs in long term assays. To address the molecular basis for this activity we analysed its interaction with putative binding partners in breast cancer cells and demonstrated relatively strong interactions with 70 kDa heat shock proteins, HSC70 and HSP70, modest interaction with the estrogen receptor, but not to BCL-2. We have created deletion and point mutations and have shown that interaction with HSC/HSP70 is mediated by the C-terminus of BAG-1. We are currently testing these mutations in our biological assays. BAG-1, HSC70 and HSP70 all relocalise from the cytoplasm to the nucleus within 4 hours after heat shock. Although the interaction between HSC/HSP70 and BAG-1 and the relocalisation of HSC/HSP70 to the nucleus are not effected by detergents, BAG-1 can be extracted from the nuclei of heat shocked cells using mild detergents. These results suggest that the relocalisation of BAG-1 is not dependent on binding to HSC70/HSP70, and that the association between BAG-1 and HSC/HSP70 may be disrupted by heat shock. We are currently analysing in detail the subcellular localisation and binding interactions of BAG-1 after heat shock, and the effect of BAG-1 overexpression on this sequestration mechanism.

### 6.8 PROLIFERATION SIGNALS IN BLADDER CARCINOMA CELLS: A NEW APPROACH

HH Seifert, S Swiatkowski, C Steinhoff, WA Schulz, Dept of Urology, Heinrich-Heine-University, Düsseldorf, Germany

To investigate which intracellular signal transduction pathway might be responsible for the increased proliferation of bladder carcinoma cells, we have chosen a new approach by comparing the activity of target promoters of several candidate pathways in bladder carcinoma cell lines and normal proliferating uroepithelial cells. Luciferase reporter plasmids whose activity was specifically dependent on either MAP kinases (SREluc), AP-1 (APIluc), β-catenin/TCF (TOPluc), or E2F (E2Fluc) were transfected into one of six well-characterized bladder carcinoma cell lines (VMCub1, VMCub2, HT1376, SD, 5637, SW1710) or into normal uroepithelial cells in primary culture stimulated to proliferation with EGF and cholera toxin. Luciferase activities were corrected for transfection efficiency and the measured activities were related to those of standard plasmids with constant constitutive activities. Controls included reporter plasmids with mutations of the specific binding sites (e.g., FLOPluc with a mutated binding site for TCF) and cotransfection of specific activators (e.g., a plasmid expressing constitutively active MEKK to activate SREluc and AP1luc).

Surprisingly, the activities of SREluc and AP1luc reflecting MAP kinase pathway activity were 3–20 fold lower in the bladder carcinoma cell lines than in several independent cultures of normal uroepithelial cells, with the highest activity observed in HT1376. Modulation of SREluc activity by EGF or serum in the carcinoma cell lines was threefold at most. Cotransfection of MEKK led to an up to 100fold induction which was most pronounced in the cell lines with the lowest basal activity. TOPluc did not show higher activities than FLOPluc in the bladder carcinoma cell lines indicating inactivity or even repression of the TCF-dependent branch of the Wnt signaling pathway. In comparison to normal uroepithelial cells, the E2F-dependent plasmid yielded significantly increased activity in HT1376 and 5637 which lack Rb protein, but was only up to 2fold more active in the other cell lines, even though three of them lack $p16^{INK4A}$. This result was confirmed with two other E2F-dependent promoters from the c-MYC and the cyclin E genes.

The pattern of proliferation signals in the nucleus of the investigated bladder carcinoma cell lines differed significantly from that observed in normal proliferating uroepithelial cells. In particular, at least two pathways often thought to be responsible for increased proliferation of tumour cells were not constitutively activated. As a clinical corollary, it is anticipated that some bladder carcinomas may not respond well to therapy using inhibition of these pathways.

### 7.1 EXPRESSION IN UVW GLIOMA CELLS OF THE NORADRENALINE TRANSPORTER GENE, DRIVEN BY THE TELOMERASE RNA PROMOTER, INDUCES ACTIVE UPTAKE OF [$^{131}$I]MIBG AND CLONOGENIC CELL KILL

M Boyd[1], RJ Mairs[1], McCluskey, S Carlin[1], SH Cunningham[1], L Wilson[1], A Livingstone[1], M Quigg, WN Keith[2], Depts of [1]Radiation & [2]Medical Oncolgy, CRC Beatson Labs, Glasgow G61 IBD, UK

**Aims** Targeted radiotherapy is the selective irradiation of tumour cells by radionuclides conjugated to tumour-seeking molecules. One promising agent is radiolabelled meta-iodobenzylguanidine (MIBG) which is actively taken up via the noradrenaline transporter (NAT), in neuroendocrine derived tumours. Our aim is to apply MIBG targeting to a wider range of tumours via transfection of the NAT transgene and to utilise the human telomerase RNA promoter (hTERC) to achieve tumour specific transgene expression.

**Methods** NAT cDNA was cloned under the control of the strong ubiquitous RSV or the human telomerase RNA promoter (hTERC). NAT expression was determined by [$^{131}$I]MIBG uptake and cell kill assessed by clonogenic assay. The capacity for upregulation of hTERC by external beam radiation was examined.

**Results and Conclusions** UVW cells transfected with RSV/NAT and hTERC/NAT, were endowed with the capacity for active uptake of [$^{131}$I] MIBG. NAT gene expression via the hTERC promoter resulted in uptake levels 70% of that achieved with the RSV promoter. We observed dose dependant cell kill of clonogens derived from [$^{131}$I]MIBG treated spheroids, with total clonogen sterilisation after administration of 5 and 7MBq/ml [$^{131}$I]MIBG to RSV/NAT/UVW and hTERC/-NAT/UVW spheroids respectively. hTERC promoter activity was upregulated 2-fold by administration of 2 Gy gamma irradiation.

These data suggest that hTERC is a strong promoter which has potential for tumour specific cancer gene therapy.

### 7.2 TRANSFECTION OF THE SODIUM IODIDE SYMPORTER GENE FOR TUMOUR TARGETING WITH RADIOIODINE AND [$^{211}$At]RADIOASTATINE

RJ Mairs[1], S Carlin[1], M Boyd[1], SH Cunningham[1], P Welsh[2] and MR Zalutsky[2], [1]Dept of Radiation Oncology, CRC Beatson Labs, Glasgow G61 1BD, Scotland, [2]Radiology Dept, Duke University, Durham, NC, USA

**Objectives** Targeted radionuclide therapy entails the delivery of radionuclides specifically to tumour cells. The most successful example is the treatment of disseminated thyroid carcinoma using sodium radioiodide (Na$^{131}$I), which is actively concentrated by the sodium (Na) iodide (I) symporter (NIS). Our aim was to transfect the NIS gene into tumour cells and to assess uptake and cell kill using the radiohalogens [$^{131}$I]iodine and [$^{211}$At]astatine.

**Methods** The human NIS gene was cloned into a plasmid vector for transfer into human glioma cells (UVW). Uptake and retention of $^{131}$I and $^{211}$ At were assessed by gamma counting after precipitation. Cell kill was determined in monolayer and spheroid culture by clonogenic assay.

**Results** Compared with UVW cells transfected with an irrelevant gene, NIS gene transfected cells exhibited 60-fold enhancement of uptake of $^{131}$I and $^{211}$At. The half time of retention of both radiohalides was 3 minutes. Uptake of $^{211}$At was completely blocked by incubation at 4°C and by treatment with 100 mM perchlorate. We observed a dose response relationship between radioactivity concentrations of $^{131}$I and clonogenic survival: 2% of clonogens survived treatment with 4MBq/ml, at which concentrations the survival of controls was > 90%.

**Conclusions** The inhibition profile demonstrated that active uptake by the transfectants of both $^{131}$I and $^{211}$At was mediated by NIS. Clonogenic survival assays confirmed the efficacy of $^{131}$I and demonstrated the potential of $^{131}$I for the treatment of cancers of non-thyroidal origin. Our preliminary results also suggest the possibility of NIS-based gene therapy for tumour targeting using the highly radiotoxic halogen [$^{211}$At]astatine.

### 7.3 TREATMENT OF LYMPHOMA WITH IRRADIATION AND ANTI-CD40 CAN INDUCE LONG-TERM PROTECTION VIA A CD8 T-CELL DEPENDENT PATHWAY

J Honeychurch[1], M Glennie[2], P Johnson[1], T Illidge[1], [1]CRC Dept. of Oncology and [2]Tenovus Research Laboratory, Cancer Sciences Division, Southampton General Hospital, Southampton University, SO16 6YD, UK

Here we describe the use of in vivo models to determine the efficacy of treating B-cell lymphoma with monoclonal antibodies (mAb) and irradiation. Two syngeneic murine lymphoma models have been employed (A31 and $BCL_1$), treated with combination total body irradiation (TBI) (1–6 Gy) and anti-CD40 mAb. When used as single therapeutic modalities, neither the TBI or mAb alone were able to extend survival by more than seven days over control cohorts. However, when TBI and anti-CD40 were used in combination, a clear radiation dose-response was seen with 100% animals treated with 6 Gy becoming long-term disease free survivors (> 100 days), versus 80% which received 5 Gy, and median survivals at lower doses were 42 days (4 Gy), 37 days (3 Gy), and 25 days (0–2 Gy) ($P < 0.01$).

Tracking experiments were conducted to follow tumour expansion and immunological response in vivo. Flow cytometric analysis revealed that in animals showing long-term protection there was a dramatic expansion of CD8 T-cells (between 3 and 10-fold), compared with those animals that received TBI alone. Simultaneous with this response, was a dramatic decrease in the number of tumour cells. When the ratio of T-cells to tumour cells was calculated, we observed a highly significant 12–16-fold greater ratio of CD8 T-cells to tumour cells in the long-term survivors treated with anti-CD40 plus higher doses of TBI, compared to those treated with mAb or TBI alone. In order to confirm that the CD8 T-cells were responsible for the observed protection, therapies were conducted in mice depleted of T-cells by anti-CD8 mAb. Here the degree of protection provided by the combination treatment was almost completely abrogated. This response is specific to TBI in combination with anti-CD40, as mAb to other targets, for example MHC class II, do not generate an immunological response, or provide protection.

We have demonstrated for the first time that irradiation and anti-CD40 mAb can have an additive therapeutic effect in vivo, and that this effect is dependent upon CD8 T-cells. These observations may have important implications for the application of RIT in the clinic.

### 7.4 CELL-BASED VECTOR DELIVERY FOR CANCER GENE THERAPY

JD Chester[1,5], RM Diaz[5], A Ruchatz[5], H Chong[5], T Clackson[2], FL Cosset[3], L Alvarez-Vallina[4,5], SJ Russell[5], KJ Harrington[5], RG Vile[5], [1]ICRF Clinical Centre, St James' Hospital, Leeds, UK; [2]Ariad Pharmaceuticals, Cambridge, MA, USA; [3]INSERM U412, Lyon, France; [4]Hospital Universitario ClÍnica Puerta de Hierro, Madrid, Spain; [5]Molecular Medicine Program, Mayo Foundation, Rochester, MN, USA

Progress towards systemic delivery of viral and non-viral vectors to treat tumours in vivo has been hindered by the limited efficiency and specificity of delivery of therapeutic genes to the tumour site.

We propose a cell-based strategy for delivery of viral vectors for tumour therapy. This strategy exploits the natural tissue tropisms of certain cells. For example lymphocytes containing a gene therapy vector might be allowed to 'home in' on areas of inflammation; macrophages to areas of hypoxia; and endothelial stem cells to areas of active angiogenesis. To provide localised, specific expression from vectors delivered in this way, we have generated a novel, retroviral gene therapy vector, which allows bi-modal (inducible and tissue-specific) regulation of transcription. Production of a retroviral genome from a plasmid vector is placed under inducible control of the rapamycin dimerisation system (Rivera et al, 1996 Nat Med 2: 1028). A second level of transcriptional regulation is provided by the presence of the tissue-specific carcinoembryonic antigen (CEA) promoter in the U3 region of the viral genome. In this way, delivery/producer cells should generate viral particles only when induced to do so by oral administration of rapamycin, after they reach the tumour. Viral particles released in this way will then be able to infect surrounding, dividing cells. However, due to the presence of the tissue-specific CEA promoter, expression of any therapeutic gene contained within the vector should only occur in colorectal cells.

As initial proof-of-principle, we show that human T cells (Jurkat), murine macrophages or murine HSC transduced with this vector can generate recombinant retroviral particles which subsequently express a retrovirally-encoded reporter (GFP) gene only in CEA-positive colorectal tumour cells (SW620, SW116, LoVo, HCT116) but not in a range of non-colorectal (HeLa, Mel624, HT1080) cells. Induction of vector production is totally dependent upon rapamycin and cell type-specificity is maintained in mixed cultures of colo-rectal and non-colorectal cells. To date, titres in the range of $10^4$–$10^5$/ml can be released from the cell vehicles.

In summary, we are developing cells and vectors which allow *in situ* production of vector at a tumour site. Exploiting the capacity of certain cell types to target particular tumour sites should increase the effective vector dose within tumours.

Part of this work has been supported by the McElwain Scholarship (awarded to JDC) from the Association of Cancer Physicians, UK.

### 7.5 A SINGLE PRE-OPERATIVE INJECTION OF Dl1502 ATTENUATED ADENOVIRUS IN INTRA-ORAL SQUAMOUS CARCINOMA.

Morley SE, [1]Brown R, [2]Kirn D, [1]Kaye S, and Soutar DS, Dept of Plastic Surgery, Canniesburn Hospital, Glasgow, [1]Dept of Medical Oncology, Glasgow University. [2]Onyx Pharmaceuticals, Richmond, California

**Objectives** dl 1502 (previously named Onyx-015) is a gene-deleted adenovirus designed to kill cancer cells that lack function of the tumour suppresser p53 protein. The virus should trigger apoptosis in healthy cells with functional p53 thereby preventing a productive infection and limiting tissue damage in non-malignant tissues. Despite previous clinical studies, little is known about spread and replication of the virus within a tumour mass or harmful effects on normal tissue. A study was devised investigating a pre-operative intra-tumoural and normal tissue injection in patients with operable intra-oral squamous carcinoma (SCC) to assess the p53 selectivity of the virus; to determine how it spread throughout tissues and to assess any damage to healthy tissues.

**Methods** 15 patients with intra-oral SCC were assigned to receive an injection of dl 1502 into each hemi-tumour 1, 3 or 14 days prior to resection, with the other half acting as a control, and an injection of dl1502 into adjacent normal tissue. P53 status of tumours was determined by gene sequencing. Following resection each hemi-tumour and the normal tissue was assayed for viral presence using in-situ hybridisation and immunohistochemisry, which was also performed to determine p21 and p53 expression and apoptosis levels.

**Results** 15 patients have been treated to date with no apparent damage to the injected normal tissue. Evidence of viral presence was found in only 3 out of 15 samples of normal tissue. Virus has been detected preferentially in p53 mutant tumour tissue with 8 out of 11 p53 mutant samples positive. Gross tumour destruction was not noted in any tumour samples but microscopic cytolysis was found in 5 tumours, all with mutant p53. p53 and p21 expression did not differ significantly between virus injected and control tumour samples. Apoptosis was noted to be higher in virus injected normal tissue than in tumour samples indicating that the virus could trigger apoptosis in cells where p53 was functional.

**Conclusion** The dl1502 adenovirus can be detected preferentially in p53 negative tumour tissues following direct intra-tumoural injection. Following injection into normal tissue the virus does not cause tissue destruction but does trigger raised apoptosis levels, as would be predicted by its proposed mechanism of action. This supports its role as a potential treatment for p53 defective tumours. As p53 is dysfunctional in at least 60% of human solid tumours the virus could potentially be useful in a wide range of human malignancy.

### 7.6 A PHASE II STUDY OF A GENE-MODIFIED VACCINIA VIRUS EXPRESSING MUCI AND IL-2 (TG1031) IN PATIENTS WITH METASTATIC BREAST CANCER

TA Plunkett, E Windmill, RB Acres[1], [1]J Taylor-Papadimitriou, DW Miles, ICRF Breast Cancer Biology Group, Guy's Hospital, UK, [1]Transgene, Strasbourg, France

The overexpression and aberrant glycosylation of MUC1 in human breast cancer results in the exposure of novel peptide epitopes and has made it a potential target for tumour immunotherapy. TG1031 is an attenuated recombinant vaccinia virus (VV) encoding both human MUC1 and the cytokine interleukin-2 (IL-2). VV was selected as the vector because of its well-documented safety profile. The VV was attenuated by both the removal of the thymidine kinase gene and by expression of IL-2, thereby minimising the risk of infection in potentially immunocompromised patients.

A single dose phase I study confirmed the tolerability and safety of TG1031, and no viral shedding was observed. An open-label randomised phase II study was designed to evaluate the anti-tumour and immunological activity of repeated administration of TG1031 to patients with MUC1-positive metastatic breast cancer. Patients received either $5 \times 10^6$ or $5 \times 10^7$ PFU by intramuscular injection at 3 week intervals for 4 cycles, and at 6 week intervals thereafter until disease progression. Twenty-five of 31 patients (85%) had already received chemotherapy for metastatic disease. Common toxicities from TG1031 included injection site reactions (20%) and transient pyrexia (20%). Two partial responses were observed (1 at each dose level; 2/3, 6%), and 15 patients (48%) had stable disease ≥4 weeks. IgG titres to VV increased in 29/30 patients tested but no significant humoral responses to the tandem repeat sequence of MUCI were demonstrated. Similarly, proliferative responses to VV were demonstrated, but there was no reactivity against the tandem repeat sequence of MUC1.

**Conclusion** Repeated administration of TG1031 was feasible and non-toxic. Although some anti-tumour activity was documented, there was no evidence of a humoral or cellular response to the tandem repeat sequence of MUC1. Further studies with less immunogenic vectors and immunological studies of regions outside the tandem repeat of MUC1 are proposed.

### 7.7 TARGETING DRUGS TO THE LIVER: CLINICAL EVALUATION OF POLYMER-BOUND DOXORUBICIN FOR THE TREATMENT OF LIVER CANCER

DH Palmer[1], LW Seymour[1], DR Ferry[1], SA Hussain[1], S Hesslewood[2], PJ Julyan[3], R Poyner[3], J Doran[1], AM Young[1], S Burtles[4], & DJ Kerr[1] on behalf of the CRC Phase I/II Clinical Trials Committee, [1]CRC Institute for Cancer Studies, University of Birmingham, B15 2TA, [2]Dept of Physics and Nuclear Medicine, City Hospital NHS Trust, Birmingham, B18 7QH, [3]Dept of Nuclear Medicine, Queen Elizabeth Hospital, University Hospital Birmingham NHS Trust, B15 2TH [4]Cancer Research Campaign Drug Development Office, Cambridge Terrace, Regent's Park, London, NW1 4JL, UK

**Purpose** The anticancer agent doxorubicin has been targeted to the hepatic asialoglycoprotein receptor (ASGPR) by linkage to N-(2-hydroxypropyl)methacrylamide copolymers bearing galactosamine. Here we report a phase I clinical and pharmacokinetic study of galactosamine-targeted poly HPNA-doxorubicin (known as PK2).

**Method** Patients with primary or secondary liver cancer for whom conventional treatment options had been exhausted were enrolled. Patients had no previous exposure to doxorubicin.

Three objectives were addressed: (i) to define toxicity associated with intravenous infusion of PK2, (ii) to determine efficiency of hepatic targeting using radio-imaging with $^{123}$I-PK2, (iii) to assess cytotoxic efficacy of PK2.

**Results** Thirty-one patients were recruited. Median age was 56 (range 19–76), 20 male, 11 female. Patients were treated in cohorts of three at a starting dose of 20 mg/m$^2$ (doxorubicin-equivalent) to a maximum tolerated dose of 160 mg/m$^2$. Dose-limiting toxicity comprised grade 3 fatigue, grade 3 mucositis, and grade 4 neutropaenia (patients 10–12). A further nineteen patients were treated with 120 mg/m$^2$. Side effects at this dose were tolerable myelosuppression, alopecia, fatigue and mucositis.

Radio-imaging confirmed significant targeting of PK2 to liver. Superimposition of SPECT and CT scans showed that the majority of radioactive polymer was associated with normal liver, with lower accumulations within hepatic tumour although this still represents substantially higher intratumoural levels of doxorubicin than expected following intravenous administration of non polymer-bound drug.

Of twenty-three patients with primary hepatocellular carcinoma, three achieved partial response and a further eleven had stable disease as demonstrated on sequential CT scans.

**Conclusions** These results indicate that galactosamine-targeted polymer conjugates can be selectively delivered to the liver following intravenous infusion. The MTD of PK2 was 160 mg/m$^2$. The recommended dose for future trials is 120 mg/m$^2$, significantly higher than the conventional dose of non polymer-bound doxorubicin. Although doxorubicin has minimal clinical activity against hepatoma, since most cytotoxic drugs have steep dose-response curves, the advantage obtained by generating significantly higher tumoural drug concentrations with the polymer make it worthwhile investigating in phase II efficacy studies.

### 7.8 NEW THALIDOMIDE ANALOGUES; ANTI-CANCER, ANTI-ANGIOGENIC AND IMMUNOSTIMULATORY.

A Dalgleish[1], J Marriott[1], A Czajka[1], I Clarke[1], K Dredge[1], G Muller[2], D Stirling[2], [1]Oncology Department, St George's Hospital Medical School, London SW17 0RE, [2]Celgene, Warren, NJ, USA

Thalidomide has been shown to be clinically active in a wide variety of clinical conditions including some tumours such as multiple myeloma (MM). In order to enhance the known anti-angiogenic properties of Thalidomide and to avoid its major side effect, a number of analogues have been developed. These fall into two major subgroups namely selective cytokine inhibitors which are phosphodiesterase type IV (PDE4) inhibitors and co-stimulatory immunomodulators. Prior to further testing, all analogues studied were shown not to induce the embryological changes induced by Thalidomide. Using rat aorta and human endothelial cell assays, we have been able to demonstrate that a number of analogues from both groups have marked anti-angiogenic activity. In addition, some of these molecules are strongly immunostimulatory, inducing lymphocyte proliferation, IL-2 production and inhibition of TNF receptor 2.

We have now shown that more recent analogues can induce tumour cell cycle arrest and apoptosis by a caspase independent mechanism with down regulation of Bcl-$X_L$ expression in pancreatic, colorectal and prostate cell lines. Two of these analogues have now entered clinical trials in patients with MM, melanoma, pancreas and renal cancers. Thalidomide has provided the framework for a series of drugs that may have a major impact on clinical cancer treatment over the next decade.

### REFERENCES

Marriott JB, Muller G, Dalgleish AG. Thalidomide as an emerging immunotherapeutic agent. *Immunology Today* 1999; **20(12):** 538–540

Marriott JB, Westby M, Cookson S, Guchian M, Goodbourn S, Muller G, Shire MG, Stirling D, Dalgleish AG, CC.3052: a water soluble analog of thalidomide and potent inhibitor of activation–Induced TNF alpha production. *J Immunol* **161(8)**: 4236–4243

Marriott JB et al. Immunomodulatory thalidomide analogues strongly inhibit the expression of TNF receptor 2 on CD4+ and CD8+ T cells during *in vitro* co-stimulation via T cell receptor: Correlation with increased activation markers and TNF-a secretion. (Submitted).

Marriott JB et al. Selective induction of tumour cell cycle arrest and apoptosis by the novel thalidomide analogue, cc-8062, is caspase independent and its associated with the down regulation of Bcl-$X_L$ expression. (Submitted).

### 8.1 A MULTICENTRE PHASE I GENE THERAPY CLINICAL TRIAL INVOLVING INTRAPERITONEAL ADMINISTRATION OF E1A-LIPID COMPLEX IN PATIENTS WITH EPITHELIAL OVERIAN CANCER OVEREXPRESSING HER-2/neu

S Madhusudan[1], N Bates[1], E Flanagan[1], Charnock FMC[1], M Gore[2], DPJ Barton[3], . Harper[4], Chris Karapetis[4], Desmond Yip[4], M Seckl[5], H Thomas[6], R Lechler[7], N Lemoine[7], M Pignatelli[8], TS Ganesan[1]. Oxford Radcliffe Hospitals[1], Oxford, Royal Marsden Hospital[2], St. George's Hospital[3], Guy's Hospital[4], Charring Cross Hospital[5], Hammersmith Hospital[7], London, Royal Surrey County Hospital[6], Guildford, Bristol Royal Infirmary[8], Bristol, UK

HER-2/neu proto-oncogene product, a transmembrane receptor tyrosine kinase, is over-expressed in 10%–30% of ovarian carcinomas and is associated with a poor prognosis. The E1A gene product of Adenovirus type 5 down regulates HER-2/neu over-expression, causing regression of tumours in animal models. Intraperitoneal administration of E1A-lipid complex is a novel gene therapy in ovarian cancer. The primary objectives of the study were to demonstrate E1A gene transduction into malignant cells after intraperitoneal administration of E1A-lipid complex, to assess HER-2/neu down regulation and to determine the maximum tolerated dose of E1A-lipid complex. Main inclusion criteria included performance status 0–3, relapse following first line therapy, tumour positive for HER-2/neu over expression (i.e ≥ 20% of cells and the intensity of reaction on immunohistochemistry), no known peritoneal loculation, normal renal, liver and coagulation profiles. Successive cohorts of at least 3 patients received ascending doses of E1A-lipid complex (1.8, 3.6, 7.2, 10.8, 14.4 mg DNA/m$^2$), to a maximum of 6 courses. Each course of therapy consisted of weekly intraperitoncal administration for 3 weeks followed by 1 week of rest. Peritoneal fluid was sampled at baseline and twice monthly for cellularity, cytology, markers (Ca 125), and biological activity in target tumour cells (E1A transduction assessed at DNA, RNA and protein levels and HER-2/neu by immunohistochemistry). A total of 15 patients were recruited (stage III-8 and stage IV-7), 14 of whom were evaluable. Median age was 57 years. Prior treatment was with platinum (80%) and taxol (53%). Patients received 1.8 mg DNA/m$^2$ (3), 3.6 mg DNA/m$^2$ (4) and 7.2 mg DNA/m$^2$ (8). One patient completed six courses of therapy; all others did not due to adverse events or disease progression. E1A gene transfer and expression in malignant cells was seen in all patients (100%). Two patients (18%) had HER-2/neu down regulation. There was no correlation between dose, E1A gene expression levels HER-2/neu expression levels, CA 125 levels nor tumour measurements. Adverse events with intraperitoneal catheter included infection and catheter blockage. Severe abdominal pain was dose-dependent and near dose-limiting toxicity was achieved at 7.2 mgs DNA/m$^2$. Intraperitoneal E1A-lipid complex gene therapy is feasible and safe. Clinical benefit will be assessed in future trials.

### 8.2 TUMOUR VASCULARITY ASSESSED USING DOPPLER ULTRASOUND PREDICTS THE RISK OF RESISTANCE TO METHOTREXATE CHEMOTHERAPY IN GESTATIONAL TROPHOBLASTIC TUMOURS

R Agarwal[1], S Strickland[1], IA McNeish[1], M Foskett[1], D Patel[2], J Boultbee[2], ES Newlands[1], MJ Seckl[1], [1]Dept of Medical Oncology and [2]Dept of Radiology, Charing Cross Hospital, London W6 8RF, UK

Tumour angiogenesis determined histopathologically is an adverse prognostic factor in several cancers. To assess tumour angiogenesis in vivo in patients with gestational trophoblastic tumors (GTT), we used Doppler ultrasound to measure the uterine artery pulsatility index (UAPI), and evaluated whether UAPI could provide independent prognostic information on the risk of resistance to methotrexate chemotherapy (MTX-R).

All patients treated for GTT between March 1994 and January 1999 had their records reviewed to determine their pre-treatment Charing Cross Hospital prognostic score (CXH-PS), uterine volume, UAPI, number of metastases and human chorionic gonadotrophin (hCG) concentration. 164 patients were included in the study, 47 of whom subsequently developed MTX-R.

UAPI, hCG, uterine volume, presence of metastases, and the overall CXH prognostic score were all predictive of MTX-R on univariate analysis. UAPI remained an independent predictor of MTX-R after multivariate analysis. The odds ratio for the risk of MTX-R in patients with a UAPI ≤ 1 compared to those with a UAPI > 1 was 2.32 (95% CI 1.14–4.7, $P = 0.02$), and after adjustment for the CXH prognostic score was 2.68 (95% CI 1.25–5.74, $P = 0.01$).

UAPI, as an indirect in vivo measure of tumour angiogenesis in GTT, is an independent predictor of response to chemotherapy.

### 8.3 AUTOLOGOUS MUC-1 PULSED DENDRITIC CELLS ARE A SAFE, FEASIBLE TREATMENT APPROACH IN PATIENTS WITH CANCER AND ARE ASSOCIATED WITH CELLULAR IMMUNE RESPONSES

F Nussey[1], M Waterfall[1], K Samuel[2], A Atkinson[2], R Leonard[1], M Turner[1,2], [1]University of Edinburgh, Dept. of Oncology, Western General Hospital, EH4 2XU, [2]Scottish National Blood Transfusion Service, Cellular Therapeutics Group, John Hughes Bennett Laboratories, Western General Hospital, Edinburgh, UK

Exploitation of the immune system in patients cancer represents a promising treatment approach. Dendritic cells (DC) are professional antigen presenting cells which are capable of inducing MHC restricted antigen specific CD4 and CD8 T cells in vitro when pulsed with tumor antigens. The function of DC in patients with malignancy is deficient and re-administration of ex-vivo cultured DC may reverse this. MUC-1 is a transmembrane glycoprotein which is aberrantly glycosylated in a number of human cancers such that an occult epitope is exposed which provides an immunotherapeutic target.

A phase 1 clinical trial of MUC-1 pulsed DC began in July 1999. 12 patients with breast, ovarian, colorectal and oesophageal cancer have been treated. 4 patients remain on follow up. Mononuclear cells were obtained from a peripheral blood donation or leucapheresis and adherent monocytes were cultured in GM-CSF and IL4 to obtain DC. These were then pulsed with a liposomal preparation of a 25-mer peptide from MUC-1 (BLP-25, Biomira Inc) and re-administered by subcutaneous injection. DC were defined as cells expressing CD1a. Patients received between $0.075 \times 10^6$ and $1.0 \times 10^6$ DC per kg body weight in one or two doses. They were followed up for toxicity, immune response and clinical effects at days 1, 7, 14, 28 and 90.

11 patients are currently assessable being more than 28 days from treatment. Minor, self limiting grade 1 toxicitites were reported by 8 patients and included fatigue and pain at the injection site. Grade 2 fatigue was seen in 2, myalgia in 1 and anaemia in 1. No grade 3 or 4 toxicities were seen. 4 patients had stable disease radiologically and clinically at 28 days, one after prior progression, but all had progressed by 3 months. Immunological effects – a small but significant increase occurred in proliferative response to the MUC-1 antigen in patients over time compared with controls ($n = 6$). There was an increase in response to repeated skin testing with PPD, a recall antigen, following the DC therapy ($n = 9$). Patients mean pre-treatment value was 3.2 mm, post treatment 13 mm ($P = 0.03$, Students t test), compared to controls ($n = 5$), who showed no response to re-testing over the same time period (initial test mean 17.6 mm, repeated test mean 23.6 mm, $P = 0.13$, Students t test).

This phase 1 trial demonstrates a safe, feasible treatment approach with biological effects in patients with a range of malignancies. Larger studies will be required to show potential therapeutic benefit. This work is supported by a grant from the Melville Trust for the Care and Cure of Cancer.

### 8.4 RAISED PLASMA HOMOCYSTEINE LEVELS IN WOMEN WITH METASTATIC BREAST CANCER

A Makris[1], RJ Burcombe[1], H Cladd[1], JM Smith[2], M Makris[2], [1]The Marie Curie Research Wing, Mount Vernon Hospital, Northwood, UK and [2]Sheffield Haemophilia and Thrombosis Centre, Royal Hallamshire Hospital, Sheffield, UK

It is well recognised that patients with malignancy have an increased risk of venous thromboembolic disease. The pathophysiology of this association has not been precisely defined. Hyperhomocysteinaemia has recently become established as one of the commonest conditions associated with venous and arterial thrombosis.

We examined the prevalence of hyperhomocysteinaemia in women with early and advanced breast cancer. Three groups of women were studied: Group 1 – healthy female controls ($n = 21$); Group 2 – early breast cancer ($n = 30$); and Group 3 – metastatic breast cancer ($n = 39$). All samples were collected prior to chemotherapy in the breast cancer patients. The homocysteine concentration was estimated in plasma using the Abbott IMx immunoassay method. Samples were separated within 1 hour of collection.

The mean (SD) plasma homocysteine levels were Group 1 = 7.9 µmol/l (1.9), Group 2 = 9.57 µmol/l (5.6) and Group 3 = 11.4 µmol/l (5.2). 35.9% of patients with metastatic and 13.3% with early breast cancer had plasma homocysteine concentrations above the upper limit of normal. Women with metastatic disease had significantly higher plasma homocysteine concentrations compared to controls ($P < 0.005$) or women with early breast cancer ($P < 0.05$). No difference was observed when women with early breast cancer were compared to controls ($P = 0.32$).

We conclude that hyperhomocysteinaemia is common in women with metastatic breast cancer but was not observed in women with early disease where homocysteine concentrations were similar to controls. This observation could explain the high rate of venous thrombosis in women with metastatic breast cancer. Since hyperhomocysteinemia is easily corrected with oral folic acid, a therapeutic trial of this drug as a thromboprophylactic agent is warranted.

### 8.5 INTRA-OPERATIVE ASSESSMENT BY OPTICAL BIOPSY FOR SENTINEL LYMPH NODE METASTASIS IN BREAST CANCER

AC Lee[1], CDO Pickard[1], MRS Keshtgar[2], SG Bown[1], GM Briggs[1], S Lakhani[3], IJ Bigio[4], PJ Ell[5], [1]National Medical Laser Centre, [2]Department of Surgery, [3]Department of Histopathology, [5]Institute of Nuclear Medicine, Royal Free and University College Medical School, University College London, Charles Bell House, 67–73 Riding House Street, London W1W 7EJ UK, [4]Department of Biomedical Engineering, University of Boston, USA

**Aims** The histological status of the axillary lymph nodes remains one of the most important prognostic indicators in breast cancer patients. It was the aim of this study to evaluate the accuracy of optical biopsy[1] (OB) as an intraoperative diagnostic tool to determine the histological status of the sentinel lymph node (SLN) in patients with invasive breast cancer.

**Procedures** Since October 1998, A total of 51 patients had been enrolled in the second phase of this study. The median age of the patients was 52 years (range, 34 to 88 years). After harvesting, the SLN was bivalved. Optical spectra were acquired using a clean probe from a number of representative points on the cut surface. The SLN was sent for histopathology.

**Results** A total of 77 SLN were biopsied from 51 patients (1.5 SLN per patient). The sensitivity of this technique was 87.1%; the specificity was 85.2%.

**Significance** Current intra-operative methods of assessing SLN for metastasis in breast cancer are fresh frozen section and imprint cytology. These techniques are operator dependent and time consuming. OB has the potential to provide an instant, non-operator dependent assessment of sentinel nodes.

**Conclusion** OB has the potential to provide instant and non-operator dependent intra-operative analysis of SLN in patients with breast cancer, which will enable the surgeon to decide on performing axillary lymph node dissection at the time of initial surgery. Sensitivity and specificity should increase as the database of correlated biopsies increase in size.

1. Bigio IJ, Bown SG and Briggs G et al. (2000) *J. Biomedical Optics* **5**(2): 221

### 8.6 EVIDENCE THAT THE CARDIOVASCULAR TOXICITY OF ANDROGEN DEPRIVATION THERAPY FOR PROSTATE CANCER MIGHT COMPROMISE A SURVIVAL BENEFIT

MD Mason[1], JC Smith[1], S Bennett[1], HG Kynaston[1], M Parmar[2], JS Davies[1], [1]University of Wales College of Medicine, Heath Park, Cardiff CF14 4XW and [2]MRC Clinical Trials Unit, London NW1 2DA, UK

One of the greatest surprises in the Prostate Cancer Trialists' Group meta-analysis of immediate versus deferred hormone therapy was that a clear benefit for immediate therapy in terms of a reduction in prostate cancer-specific mortality did not translate into an overall survival benefit. We reasoned that this might be due to an, as yet, undocumented detrimental effect of androgen deprivation on survival, and that cardiovascular morbidity was a likely mechanism. Were this true, amelioration of this effect might yield survival benefits for early androgen deprivation, at best to an extent comparable to the benefits of adjuvant therapy for breast cancer. In order to test this, we studied the effects of androgen deprivation therapy on large artery stiffness in 22 prostate cancer patients (mean age 67±8 yrs) over a 6 month period. Arterial stiffness was assessed using Pulse Wave Analysis, a technique that measures peripheral arterial pressure waveforms and generates corresponding central aortic waveforms. This allows determination of the augmentation of central pressure resulting from wave reflection and the Augmentation Index, a measure of large artery stiffness. Body compositional changes were assessed using Bioelectrical Impedance Analysis. Fasting lipids, glucose, insulin and testosterone were measured. Following a 3 month treatment period, the Augmentation Index increased from 24 ± 6 (mean ± SD) at baseline to 29 ± 9% ($P$ = 0.003) despite no change in peripheral blood pressure. Timing of wave reflection was reduced from 137 ± 7 to 129 ± 10 ms ($P$ = 0.003). Fat mass increased from 20.2 ± 9.4 to 21.9 ± 9.6 kg ($P$ = 0.008) whilst lean body mass decreased from 63.2 ± 6.8 to 61.5 ± 6.0 kg ($P$ = 0.016). There were no changes in lipids or glucose during treatment. Serum insulin rose from 11.8[5.6 – 49.1] (median[range]) to 15.1[7.3–83.2]mU/L at 1 month ($P$ = 0.021) and to 19.3[0 – 85.0]mU/L by 3 months ($P$ = 0.020). There was a correlation between the changes in fat mass and insulin concentration over the 3 month period ($r$ = 0.56, $P$ = 0.013). In a sub-group of patients whose treatment was discontinued after 3 months, the Augmentation Index decreased from 31±7 at 3 months to 29 ± 5% by 6 months in contrast to patients receiving continuing treatment in whom the Augmentation Index remained elevated at 6 months compared with baseline ($P$ = 0.043). These data suggest that androgen deprivation results in large artery stiffening and reduced insulin sensitivity, both established markers of increased cardiovascular risk. We are now confirming these results further in a larger prospective study.

### 8.7 RESTORATION OF OVARIAN FUNCTION AFTER REIMPLANTATION OF AUTOLOGOUS CRYOPRESERVED OVARIAN CORTICAL STRIPS (OCS) FOLLOWING HIGH DOSE CHEMOTHERAPY FOR LYMPHOMA

JA Radford[1], DR Brison[2], SA Russell[2], M Harris[1], AJ Watson[1], JA Clayton[1], RG Gosden[3], SM Shalet[1], BA Lieberman[2], [1]Christie Hospital, Manchester, UK, [2]St Mary's Hospital, Manchester, UK, [3]McGill University, Montreal, Canada

Since February 1996, 16 women (median age 24 years, range 17–36) have undergone unilateral laparoscopic oophorectomy shortly before receiving high dose chemotherapy (HDCT) for lymphoma ($n$ = 14) or acute lymphoblastic leukaemia ($n$ = 2). The surgical procedures were well tolerated and without complication. In each case the cortex was stripped from the ovary *en bloc*, flattened, trimmed and cut into strips (OCS) approximately 1 × 0.5 cm (median 7 OCS per ovary, range 5–10). Following equilibration in cryoprotectant (1.5 molar DMSO), the OCS were placed in individual vials, cooled in a programmable rate freezer with seeding at –9 ˚C and stored in liquid nitrogen. Since HDCT 6 patients (pts) have died of disease and 10 are alive and disease-free. One of the latter (now aged 39, received HDCT August 1998) opted for reimplantation of OCS and hormone replacement therapy (HRT) was discontinued in mid-November 1999. By assaying serum sex steroids, ovarian failure was confirmed and laparoscopic reimplantation of 2 OCS was performed in March 2000. Each OCS was removed from liquid nitrogen and kept at room temperature for 30 seconds before being plunged into a 37˚C water bath for approximately 1 minute. DMSO was removed by three, 5 minute rolling washes in 10 ml of Liebovitz medium. The OCS were trimmed and then placed in sterile medium before being transferred to theatre where 1 OCS was grafted onto the ovarian pedicle (oophorectomised side) and another onto the remaining ovary (non-oophorectomised side). Seven months following reimplantation of OCS (mid October 2000) the patient became free of hot flushes and serum oestradiol became detectable. The LH and FSH have since fallen to 19 iu/L and 41 iu/L respectively (previously 84 iu/L and 110 iu/L). Pelvic ultrasonography performed 22/11/2000 (oestradiol 352 pmol/L) showed an endometrial thickness of 11 mm, a residual left ovary with an approximate volume of 2 mls containing no follicles and to the right of the midline (oophorectomised side) a follicular structure with an approximate volume of 6 mls and containing a dominant 2 cm follicle. On 29/11/00 (oestradiol 120 pmol/l) the left ovary remained small with no evidence of any follicular development and the right sided cyst was no longer visible. The patient subsequently menstruated.

Although this is a preliminary report it appears that orthotopic reimplantation of frozen thawed OCS is an effective technique for restoring ovarian function in women treated with sterilising chemotherapy for lymphoma.

### 8.8 FERTILITY AND QUALITY OF LIFE AFTER TREATMENT FOR TESTICULAR CANCER

RA Huddart[1,2], A Norman[1], C Moynihan[2], D Coward[2], J Nicholls[2], G Jay[2], M. Shahidi[2], A Horwich[1,2], D Dearnaley[1,2], The Royal Marsden Hospital[1] and The Institute of Cancer Research[2], Sutton, Surrey, SM2 5PT, UK

Modern treatments cure most testicular cancer patients so an important goal is to minimise toxicity. We have undertaken a cross-sectional study to evaluate the quality of life (Qol) of long term survivors of testicular cancer. 654 patients treated between 1982 and 1992 completed the EORTC Qly-C-30(QC30) questionnaire, the associated testicular cancer specific module and a general health and fertility questionnaire. Patients have been subdivided according to treatment received: orchidectomy either alone (surveillance, S), with chemotherapy only (C), radiotherapy only (R), or both chemotherapy and radiotherapy (C/RT).

Only 221 (30%) patients reported attempting conception after treatment. When attempted, the success rate was high with 178 (81%) reporting success. A further 12 patients (5.4%) were successful after infertility treatment but 43 (19%) were unsuccessful, (with or without fertility treatment). There was a trend to lower success rate after C (75%) ($P$ = 0.133) compared to S (85%).

Overall Qol was good with mean scores in the all domains of the QC30 in the range of 83–95; equivalent to or in excess of both pre-treatment testicular patients and normal population reference scores. 53% of patients reported anxiety regarding recurrence which was moderate/severe in 11% especially in patients receiving treatment (C 13%, C/RT 10%, RT 13%, S 5%). Treated patients also had a higher chance of impaired social functioning (S 95, RT 92 ($P$ = 0.24), C/RT 89 ($P$ = 0.003), C 92 ($P$ = 0.056) ). 42% of patients reported difficulties with work or obtaining insurance mortgages especially after C (S 37% C 46%). 18% felt their disease had affected their relationship (especially after chemotherapy C 19% ($P$ = 0.175), C/RT 26% ($P$ = 0.022) v S 14%).

Compared to S, patients after C were more likely to report tingling ($P$ = 0.001), pale cold hands ($P$ = 0.001), ringing in the ears ($P$ = 0.05); dyspnoea ($P$ = 0.05) and worries about fathering ($P$ = 0.009) and in the C/RT upset about hair loss ($P$ = 0.017) and less interest in sex ($P$ = 0.01). R alone was associated with reduced sexual activity/enjoyment ($P$ = 0.05)

In summary the majority of long term survivors have a good quality of life. Most patients retain their fertility but the risk of infertility is increased by chemotherapy. However, patients can suffer from long term effects of treatment and psychosocial sequelae including difficulties in obtaining insurance.

### 9.1 ICON1: A RANDOMISED TRIAL OF IMMEDIATE PLATINUM-BASED CHEMOTHERAPY AGAINST CHEMOTHERAPY DELAYED UNTIL INDICATED IN WOMEN WITH OVARIAN CANCER

D. Guthrie on behalf of the ICON Collaborators, MRC Clinical Trials Unit, 222 Euston Road, London NW1 2DA, UK

**Background** A series of meta-analyses of randomised controlled trials raised the question of whether adjuvant chemotherapy prolongs disease-free survival in women with early stage epithelial ovarian cancer.

**Method** We carried out an international, multicentre, randomised trial to compare immediate platinum-based chemotherapy against chemotherapy delayed until indicated, in women with ovarian cancer for whom doctors were uncertain if chemotherapy was required immediately after optimal primary surgery.

**Findings** The first results will be presented at the EORTC/BGCS/UKCCCR/MRC Collaborators' Meeting on Gynaecological Cancer in April 2001, and therefore results by treatment arm are not presented here. 447 patients were randomised from 91 centres in five countries; 241 to immediate treatment and 236 to delayed treatment. The median age was 55 years with over 80% patients being FIGO stage 1. The major histological cell types were serous (30%), mucinous (21%), endometrioid (21%) and clear cell (14%). Differentiation of disease was classified as poor in 27% of patients, intermediate in 41%, and well in 32% of patients. The patient characteristics were similar in both treatment groups. With over 3 years median follow-up for survivors, it was estimated that the 3 year progression-free survival was 78%, and overall survival was 85%.

It is hoped that the data from ICON1 and ACTION, a comparable trial launched by the EORTC and run in synchrony with ICON1, can be combined.

### 9.2 AIM HIGH – ADJUVANT INTERFERON IN MELANOMA (HIGH RISK): NO CONFIRMATION YET THAT LOW DOSE INTERFERON IS OF BENEFIT

B W Hancock[1], LA Turner[1], K Wheatley[2], G Harrison[3], M Gore[4], [1]Weston Park Hospital, Sheffield, [2]University of Birmingham, [3]University of Oxford, [4]Royal Marsden Hospital, London, UK

In the UK, the mortality rate from melanoma has doubled over the past 20 years. This currently represents about 2% of all new cases of cancer and 1% of all cancer deaths[1]. It has been shown that Interferon can be an effective palliative treatment in metastatic melanoma. There is early evidence that treatment prolongs disease free survival and may have an effect on overall survival[2,3]. The primary objective of this trial was to determine the effects of Interferon alpha-2a with observation alone on overall survival (OS) and recurrence-free survival (RFS) of patients with high risk malignant melanoma. Secondary objectives were to study the interaction of Interferon alpha-2a with age and sex, to document the side effects of long term administration of Interferon and evaluate the economic implications to the health service should Interferon prove effective in this group of patients. Between 3 October 1995 and 22 December 2000, a total of 674 patients were recruited from 37 centres in the UK. 337 patients were treated with Interferon alpha 2a 3 million units three times a week until recurrence or for two years and 337 patients with observation alone. The arms of the study were well balanced for age, sex and type of disease. 130 had a primary tumour ≥ 4 mm Breslow thickness, 74 non-nodal superficial regional recurrence, 85 regional lymph nodes resected at presentation and 385 regional lymph nodes resected at recurrence. Median follow up is 489 days (range 2–1885 days). The OS and RFS at four years for all patients are 51(se3.0)% and 31(se2.6)% respectively. At four years there was no significant difference in OS or RFS between the Interferon treated and control arms (52 se4.0% vs 50 se4.4%, $P = 1.0$, and 33 se3.6% vs 29 se3.7%, $P = 0.2$, respectively). Male sex ($P = 0.04$) and regional lymph node involvement ($P = 0.002$) were statistically significant adverse features for OS. Subgroup analysis by age, sex and disease has not yet shown any significant differences between Interferon treated and control groups in either OS or RFS. Although preliminary meta-analysis showed a statistically significant advantage for Interferon regardless of dose[1], these preliminary results from AIM HIGH do not yet confirm that extended duration low dose Interferon is better than observation alone in the initial treatment of completely resected high risk malignant melanoma.

1. BW Hancock et al, *Cancer Treatment Reviews* 2000; **26**: 81
2. JM Kirkwood et al, *J Clin Oncol* 1996; **14**: 7.
3. JJ Grob et al, *Lancet* 1998; **351**: 1905

### 9.3 AN UPDATE REPORT ON THE MRC CR07 TRIAL

D Sebag-Montefiore on behalf of the MRC Colorectal Cancer Group and all the CR07 participants. Cancer Division, MRC Clinical Trials Unit, 222 Euston Road, London, UK

This randomised trial compares 25 Gy pre-operative radiotherapy (RT) and selective post-operative chemo-radiotherapy (45 Gy with synchronous 5 FU) in rectal cancer. The trial opened in March 1998 and by the end of 2000 468 patients have been accrued from 39 centres. Pre-treatment characteristics: male 70%, median age 70 years, distance from the tumour to the anal verge ≤12 cm 95%.

Overall 59% of patients have had an anterior resection (AR) and 35% APER. Total mesorectal excision (TME) surgery is not mandatory; however in the surgeon's opinion TME was intended and achieved in 90% of patients. Wound infections (16%), non-healing perineum (12%) and chest infection (6%) are the main post-op morbidities. The anastomotic leak rate is 12% in patients undergoing an AR.

Pathologists have reported the quality of the mesorectum on the specimen as moderate or good in 84%, and that there were 5% pT1, 27% pT2, 61% pT3 and 7% pT4. 84% of patients have had a complete resection at all margins. The median number of lymph nodes sampled is 11, and 44% of patients have positive nodes. The 30-day post-op mortality is 3%.

In the pre-op RT group 93% of patients have received 25 Gy/5f as prescribed. Median time from randomisation to start of RT is 17 days and from end of radiotherapy to surgery 5 days. In the post-op group the median time from randomisation to surgery is 17 days, and 76% of the patients with a positive circumferential resection margin (CRM) have received chemo-radiotherapy (a further 14% received RT alone).

This trial will complement the results of the Dutch TME trial.

### 9.4 IS SOME NEUTROPENIA GOOD FOR YOU? A SINGLE CENTER EXPERIENCE OF ADJUVANT CMF IN 681 CASES OF EARLY BREAST CANCER

C Massie, G Kerr, RCF Leonard, DA Cameron
On behalf of the Edinburgh Breast Group, Dept. Oncology and Breast Surgery, WGH Edinburgh, UK

An audit of women receiving adjuvant i.v. CMF chemotherapy for early stage breast cancer identified over 700 patients who were treated between 1984 and 1998 by the Edinburgh Breast Group. The casenotes of 681 patients have been reviewed and the results are presented here.

**Results: Patients** Because of changing selection policies more than 50% of patients were treated during the last 3 years of the period. Median age was 47 years, range 26–86 years. 13.2% of patients were aged 60 or over. The majority of patients (67%) presented with clinical stage 2 disease. Median pathological tumour size was 2 cm. 68% had involved lymph nodes. 52.7% had high grade tumours.

**Results: Treatment** 92.7% of patients completed 6 courses. 53% of patients had a treatment delay and 8% a dose reduction due to toxicity. Dose reductions were twice as common in patients aged 60 or over. Dose intensity ranged from 64% to 105% of planned, median 85%. 549 patients received adjuvant radiotherapy, intercalated in 461.

**Results: Outcome** There were no treatment related deaths and only 8 non breast cancer deaths. 5 year cause specific survival was 71.5%; 68.7% for stage 2 disease. Node negative patients had a 5 year cause specific survival of 85.9%. Heavy node involvement was associated with poor survival. 46% of patients had grade 2 or 3 neutropenia which was associated with better long-term survival (80.5% at 5 years) than grades 0, 1 or 4 (63.9% at 5 years), $P = 0.0001$. Grade 4 neutropenia did not lead to a reduction in the number of treatment cycles. Patients who completed less than 4 courses of CMF had a significantly poorer survival than those who completed 4 or more. Dose intensity appeared to have no effect on survival on univariate analysis.

**Conclusions** In 681 reviewed cases the usual prognostic factors have been shown to be associated with survival, outside the trial setting. The data suggest that achieving a planned dose of CMF may be less important than planning an appropriate dose.

### 9.5 THE DEVELOPMENT OF CONSENSUS GUIDELINES FOR THE MANAGEMENT OF CNS TUMOURS AS A RESULT OF A NATIONAL SURVEY, WORKSHOP AND LITERATURE REVIEW

G Gerrard[1], K Dyker[1], D Levy[2], [1]Dept. of Clinical Oncology, Cookridge, Leeds, LS16 6QB, [2]Weston Park Hospital, Sheffield, S10 2SJ, UK

This study aimed to encourage collaboration amongst clinicians to ascertain regional variations in treatment of CNS tumours before comparing inter-regional survival figures in a national database. It also aimed to identify areas of treatment divergence to stimulate debate, education and research and to create treatment guidelines.

In November 1999, 54 clinical oncologists with an interest in primary CNS tumours were sent a questionnaire regarding treatment issues for high and low grade gliomas, meningiomas, pituitary adenomas, craniopharyngiomas, clivus chordomas, brain metastases and temozolomide.

In adult high-grade gliomas 78% (32/41) prescribe 60 Gy in 30 fractions over 6 weeks to young, good performance status patients. 66% (27/41) use CT planning. 95% (39/41) will treat patients over 70 years of age with the above regime if they are of good performance status. If they are of poor performance status, 59% (24/41) use 30 Gy in 6 fractions over 2 weeks, but there are another 10 fractionation regimes used in the UK.

97% (37/38) give craniospinal radiotherapy (CSRT) for high grade ependymomas with CSF seeding and 61% (23/38) give it if no CSF seeding. 95% (36/39) would treat lowgrade ependymomas with CSRT only if they had CSF seeding.

For adult low-grade gliomas 72% (29/40) reserve treatment for clinical progression. 61% (25/41) give 54Gy in 30 fractions, with another 8 dose regimes in use in the UK. 80% (33/41) treat benign meningiomas when indicated and 27% (11/40) give radiotherapy to all atypical meningiomas. The commonest regime for all, including malignant meningiomas is 54Gy in 30 fractions.

In pituitary adenomas, 61% (25/41) use CT planning, 95% (39/41) giving 45Gy in 25 fractions. 95% (36/38) give radiotherapy for all incompletely excised craniopharyngiomas, 69% (27/39) using CT planning. 78% (31/40) give 50 Gy in 30 fractions. Proton treatment in Paris remains a controversial issue for clivus chordoma.

In elderly, poor performance status patients 65% (24/37) treat some with whole brain radiotherapy (WBRT) for brain metastases. 85% (35/41) would give WBRT after resection of a solitary metastasis while 15% (6/41) would observe such patients.

47% (19/40) give temozolamide as second line chemotherapy for recurrent glioblastoma 40% (16/40) do not use it as there is no local funding or that they feel it is no better than standard.

**9.5 Cont'd**

For most CNS primaries, the majority of clinicians use similar regimes. The most variation occurs in treatment of low- grade gliomas and elderly patients with high-grade gliomas or brain metastases. The 54 participants will be informed of the results and alternative regimes. The plan is to hold a workshop for discussion in order to establish treatment guidelines. As the clinicians will be involved in this process, it may encourage them to follow them, rather than have them imposed from above.

### 9.6 EARLY RESULTS FROM THE UK HEAD AND NECK (UKHAN) TRIAL: THE ROLE OF CHEMOTHERAPY IN PRIMARY MANAGEMENT OF ADVANCED HEAD AND NECK CANCER

JS Tobias[1], KM Monson[1], J Glaholm[2], RH MacDougall[3], NK Gupta[4], J Peto[5], W Sawyer[1], J Houghton[1] on behalf of the UKHAN Trial Group, [1]CRC & UCL Cancer Trials Centre, London NW1 2ND, [2]Birmingham Oncology Centre, B15 2TH, [3]Western General Infirmary, EH4 2XU, [4]Christie Hospital, M20 4BX, [5]ICR, Sutton SM2 5NG, UK

The UKHAN trial was designed as a large-scale single, pragmatic study investigating the effect of adding chemotherapy to standard radical treatments for advanced head and neck cancer. In order to make participation simple and ensure the results would be widely applicable, outpatient chemotherapy (CT) protocols were chosen and all standard radiotherapy regiments (RT) were accepted, if approved by the working party.

Patients presenting with UICC stage II, III & IV, squamous cell carcinoma of the head and neck, who were suitable for RT to be given either as definitive radical therapy or as post- operative treatment, were considered eligible and were approached for consent. Patients with distant metastases were excluded. Non-surgical patients were randomised to one of four arms: [1] RT alone.[2] RT with 2 courses of simultaneous CT administered days 1 & 14 of RT (SIM CT). [3] RT with 2 courses of subsequent CT administered 14 & 28 days after completion of RT (SUB CT). [4] RT with both SIM CT & SUB CT (4 courses). Surgical patients requiring post-operative RT were randomised to arms [1] & [2] only. Chemotherapy was given either as single agent methotrexate or combination VBMF (vincristine, bleomycin, methotrexate, 5-fluorouracil). Participating clinicians selected one of these regimens consistently throughout the study period to all patients randomised to receive CT.

Between 1990–2000 the trial accured 970 patients. At closure (July 2000) an interim analysis was performed on 947 patients followed for at least 6 months (median follow up 3.5 years). Clinical response was assessed at six months from randomisation; 73% of the SIM CT patients were disease-free compared with 69% of the RT alone (non-significant). These patients also showed an improved event-free survival (RR = 0.77, 95% CI 0.64–0.93) but no significant improvement in overall survival has been demonstrated. No advantage has been shown for SIM CT in the surgical group ($n$ = 243) or for patients randomised to SUB CT. Treatment related deaths occurred in <2% of the total trial cohort.

Early analysis of this large study suggests that non-platinum based chemotherapy given simultaneously with standard RT confers a benefit to patients that is similar to regimens using cisplatinum [see Pignon et al, *Lancet* 2000; **355**: 949]. The regimens used in UKHAN are inexpensive, well tolerated and widely acceptable, even in this unfit group of patients with advanced head and neck cancer.

### 9.7 FACTORS INFLUENCING THE USE OF THORACIC RADIOTHERAPY (TRT) IN LUNG CANCER PATIENTS IN SCOTLAND

SC Erridge[1], CS Thomson[2], J Davidson[2], RD Jones[3], A Price[1] on behalf of the Scottish Lung cancer Trials Group and Scottish Cancer Therapy Network. [1]Department of Oncology, Western General Hospital, Edinburgh, [2]Scottish Cancer Intelligence Unit, ISD Scotland, Edinburgh, [3]Department of Oncology, Beatson Oncology Centre, Glasgow, UK

**Background** Outcomes of patients with lung cancer in Scotland are poor in comparison with other countries.

**Aims** To determine the frequency of delivery of TRT to patients with lung cancer in Scotland in 1995, and identify patient, disease and process variables affecting the probability of receiving TRT.

**Methods** Retrospective case note audit of all patients with lung cancer diagnosed in Scotland in 1995.

**Results** 1188 (30.8%) of 3855 patients diagnosed with lung cancer in Scotland in 1995 for whom the medical records could be traced received TRT. In those who did not have small cell lung cancer, multivariate analysis indicated that diagnosis by a lung cancer specialist, clinical extent of disease and microscopic verification of cancer (all $P < 0.0001$) and age ($P < 0.0005$) were associated with an increased chance of receiving TRT. There was also a wide variation between different Health Boards of residence in the proportion of patients receiving TRT ($P < 0.0001$). There was no association between the presence of local symptoms (cough, chest pain or haemoptysis) and the probability of delivery of TRT.

Of 351 patients with limited stage small cell lung cancer, 51 (14.5%) received chemotherapy and TRT, and 19 (5.4%) chemotherapy and cranial irradiation.

**Conclusions** TRT was delivered to fewer than one third of lung cancer patients in Scotland in 1995. This is lower than in other international audits. The chance of receiving TRT seemed to be associated with service issues rather than clinical need.

### 9.8 ADJUVANT CHEMOTHERAPY IN EARLY BREAST CANCER: WHAT DO PATIENTS UNDERSTAND?

HE Innes[1], C Holcombe[2], SM O'Reilly[1], [1]Clatterbridge Centre for Oncology, Merseyside CH63 4JY and [2]Royal Liverpool University Hospital, Liverpool L7 8XP, UK

Women with early breast cancer, even those with relatively low risk of recurrence, are increasingly offered adjuvant chemotherapy. Patients are now being encouraged to participate in the decision whether to have this treatment. To do this effectively they need to be equipped with adequate information. This study explores the knowledge and understanding of a group of women who have completed adjuvant chemotherapy. We sent questionnaires to all 249 surviving patients who received adjuvant chemotherapy between 6/95 and 6/99 at the Royal Liverpool University Hospital, all treated by the same Medical Oncologist. All had been told the size, grade and no. of involved nodes and had been advised of potential toxicity and likely prognosis.

182 patients replied (73.1%), median age at diagnosis 47.5 (29 to 69). 22.5% were educated beyond O level. Most patients felt that the level of information was 'about right' regarding details of tumour (66.3%), treatment options (85.8%) and toxicity of chemotherapy (84.5%). On risk of recurrence 48.8% felt that information was 'about right', 50.6% 'too little' and 0.6% 'too much'. 98 (53.8%) of respondents remembered being told the risk of recurrence. 150 (82.4%) of respondents gave their own estimates of their risk of recurrence at 5y. Patients' estimates of recurrence risk and their expectations of the benefits of chemotherapy were compared with their actual risks as determined by the Early Breast Cancer Trialists' Collaborative Group overview.

| Age | Nodal status | Est. % risk of rec. without chemo at 5 y (median) | Actual % risk of rec. without chemo at 5 y | Est. % risk of rec. with chemo at 5 y (median) | Actual % risk of rec. with chemo at 5 y |
|---|---|---|---|---|---|
| < 50 years | Node –ve | 50 | 34.1 | 15 | 24.7 |
| *N* = 100 | Node +ve | 60 | 58.1 | 20 | 42.9 |
| 50–69 years | Node –ve | 42.5 | 29.7 | 12.5 | 23.4 |
| *n* = 50 | Node +ve | 62.5 | 46.7 | 20 | 40 |

**9.5 Cont'd**

When asked what degree of benefit they felt would make chemotherapy worthwhile, >70% would accept a reduction in the risk of recurrence at 5 y of ≤5% (range 0.5–60%). In conclusion many patients overestimate both the baseline risk of recurrence and the potential benefit of chemotherapy. However, the majority of these patients, all of whom have experienced adjuvant chemotherapy, would be prepared to accept similar treatment again for relatively modest benefit. On the basis of these results we have changed the written and verbal information given to patients in order to improve understanding and enable more informed participation in the decision making process.

### 10.1 EPIGENETICS OF WILMS' TUMOUR

KW Brown, S Jackson, K Moorwood, A Hancock, A Dallosso, KTA Malik, CLIC Research Unit, Dept. Pathology & Microbiology, School of Medical Sciences, University Walk, Bristol BS8 1TD, UK

Despite intensive investigation of the genetics of Wilms' tumour (WT), the molecular pathogenesis of many cases remains unclear. We have therefore been studying novel epigenetic changes in WT, to determine whether these are important in WT development. Specifically, we have examined changes in DNA methylation that are known to affect gene expression. In the WT 11p13 tumour suppressor gene *WT1*, we have shown differential methylation of the antisense regulatory region (ARR) in normal kidney, where one allele is hypermethylated and the other hypomethylated. In 80% of WTs that lack 11p LOH, both alleles of the *WT1* ARR are hypomethylated, and a similar change is seen in premalignant nephrogenic rests (NRs). The differential methylation in normal kidney is associated with genomic imprinting of the *WT1* antisense transcript, leading to monoallelic expression from the paternal allele. In WTs and NRs, imprinting is relaxed, with biallelic expression of antisense RNA. We have previously shown that in vitro expression of antisense RNA affects WT1 protein levels, and so loss of imprinting of the *WT1* ARR may be an important step in the development of WT.

Using an array-based screening technique, we have detected altered methylation of a number of other loci in WT. One of these shows differential methylation in normal kidney and hypomethylation in WT, like the *WT1* ARR. This locus maps to chromosome 12q, an area frequently involved in numerical cytogenetic changes in WT.

These results indicate that epigenetic alterations are a common early change in WT and may play a vital part in the molecular pathogenesis of WT.

This work was supported by the Cancer & Leukaemia in Childhood charity.

Malik K et al 2000 Identification of differential methylation of the WT1 antisense regulatory region and relaxation of imprinting in Wilms' tumour. *Cancer Res* **60**: 2356–2360

Malik K & Brown KW 2000 Epigenetic gene deregulation in cancer. *Br Cancer* **83**: 1583–1588

### 10.2 GENOMIC IMBALANCES IN PAEDIATRIC EPENDYMOMAS; A UNITED KINGDOM CHILDREN'S CANCER STUDY GROUP (UKCCSG) APPROVED STUDY

SA Dyer[1], EJ Prebble[1], EV Davison[1], DW Ellison[2], RG Grundy[3], [1]Regional Genetics Laboratory, Birmingham Women's Hospital, Edgbaston, Birmingham, B15 2TG, [2]Cancer Research Unit, University of Newcastle, Newcastle, [3]Institute of Child Health, University of Birmingham, Whittle Street, Birmingham, B4 6NH, UK

Ependymomas are the third most common primary brain tumour of childhood accounting for 10–15% of all tumours in this age group. Analysis of the traditional clinico-pathological variables of histology, age and site has yielded conflicting results and currently there are no clear prognostic factors for childhood ependymomas. Part of the reason for this relates to our poor understanding of the biology of these tumours.

We have initiated a large, retrospective comparative genomic hybridisation (CGH) study of 70 formalin fixed paraffin embedded (FFPE) ependymomas. The use of FFPE-CGH was validated in our laboratory using 15 fresh/FFPE ependymoma pairs. Complete correlation of paired fresh/FFPE tumour CGH profiles was observed.

To date, we have analysed 33 primary and 9 recurrent FFPE ependymal tumours collected from 38 children. Genomic imbalances were observed in 20/33 (61%) primary ependymomas and 8/9 (89 %) recurrent tumours. The mean number of imbalances for both primary and recurrent tumours was 2.7. Whole chromosome imbalances were more common in the primary tumours, whereas partial gains and losses predominated in the recurrent tumours. The most common imbalances observed in primary ependymomas were gain of 1q (27%), gain of 9p (24%), loss of 17p (12%) and loss of 6q (9%). The recurrent ependymomas most frequently exhibited gain of 1q (67%) and loss of 6q (22 %).

CGH analysis of the remaining 28 FFPE ependymoma samples is in progress and results from the complete series will be correlated with clinical details.

### 10.3 PAX3-FKHR INDUCES MORPHOLOGICAL CHANGES AND ENHANCES CELLULAR PROLIFERATION AND INVASION IN RHABDOMYOSARCOMA

J Anderson[1], A Ramsay, D Henderson, [1]Unit of Molecular Haematology, and Department of Haematology and Oncology, [2]Department of Histopathology, [3]Neurodevelopment Unit, Institute of Child Health and Great Ormond Street Hospital for Children, London, UK

Alveolar rhabdomyosarcoma (ARMS) was originally distinguished from other subtypes of rhabdomyosarcoma on the basis of its morphological appearance. Since the recognition of this entity, it has been shown to have a poorer prognosis and to be consistently associated with the characteristic translocations t(2;13)(q35;q14) and t(1;13)(p36;q14), which encode for the PAX3-FKHR and PAX7-FKHR fusion oncoproteins respectively. We have investigated the relationship between PAX3-FKHR expression and ARMS histogenesis by correlating their phenotype in primary tumors and by analyzing the effects of ectopically expressed PAX3-FKHR on embryonal rhabdomyosarcoma (ERMS) cell lines and developing myoblasts in transgenic mice.

In a previous blinded histological review of discrepant primary tumors in which there was PAX3-FKHR expression but ERMS histology, we found small areas of alveolar histology in 6 out of 11 cases. This suggests that histology alone may underrepresent the association between PAX3-FKHR and ARMS, and we proceeded to investigate this link by examining the effect of ectopic PAX3-FKHR expression on ERMS cells. Two ERMS cell lines, RD and HX170C were stably transfected with a PAX3-FKHR expression construct. In cloned transfectants derived from both cell lines PAX3-FKHR expression resulted in increased proliferative rate in vitro and promoted cell growth in the absence of added growth factors. Tumors that formed as xenografts in immunodeficient mice were faster growing, more locally invasive and had a denser, more pleomorphic architecture. The characteristic clefts and alveolar spaces of ARMS however were not seen. The denser cellular architecture suggests increased cellular adhesion. PAX3-FKHR expression by itself did not result in greater metastatic spread. In contrast, tumors grown as xenografts from individual clones derived from several ARMS cell lines showed all the classical morphological features of ARMS suggesting divergence in vivo from precursor cells propagated in culture.

Because of the possible origin of rhabdomyosarcoma cells in early development, we chose to investigate further the role of PAX3-FKHR on migration and growth by assessing the effect of its forced expression in developing myoblasts. We therefore generated transgenic mice with PAX3-FKHR expression controlled by the murine MyoD promoter. Embryos demonstrate both failure of normal myogenesis and aberrant migration of myoblasts, recapitulaing some of the features of ARMS. Ongoing studies are employing tamoxifen-inducible regulatory PAX3-FKHR cellular systems and gene array technology to identify the genes regulated in ERMS cells that are responsible for the more malignant phenotype.

### 10.4 EXPRESSION PROFILE OF *ETV6, CBFA2* AND *ETV6-CBFA2* IN CHILDHOOD ALL AND AML

N Patel[1], LK Goff[2], LK Jones[1], T Clark[3], N Foot[3], D Lillington[3], S Hing[4], K Pritchard-Jones[4], V Saha[1], [1]ICRF Children's Cancer Group, [2]Dept of Medical Oncology, St Bartholomew's and The Royal London School of Medicine and Dentistry, London EC1M 6BQ, [3]Centre of Statistics in Medicine, Institute of Health Sciences, Oxford OX3 7LF, [4]Dept of Paediatric Oncology, Institute of Cancer Research/Royal Marsden NHS Trust, Surrey SM2 5PT, UK

The transcription factors, *ETV6* and *CBFA2* are essential in the development of normal haematopoiesis. In about 25% of childhood ALL, these two genes are rearranged to form the *ETV6-CBFA2* chimeric gene. This study used real-time PCR (RQ-RTPCR) to investigate the expression pattern of *ETV6, CBFA2* and *ETV6-CBFA2* in three groups of patients: *ETV6-CBFA2* Positive ALL (Group A), *ETV6-CBFA2* Negative ALL (Group B), and AML (Group C) patients.

**Method** Bone marrow samples were taken in disease (at presentation or at relapse) and in remission states from 16 Group A, 13 Group B and 9 Group C patients. Total RNA was extracted from mononuclear cells using the RNAzol-B method, reverse transcribed into cDNA with M-MLV reverse transcriptase and random hexamers. All samples were analysed in parallel with $\beta_2 M$ as the internal control gene by RQ-RTPCR. The cycle threshold ($C_T$) value was determined from the amplification plot for the individual gene and then subtracted from the $\beta_2 M$ $C_T$ value to obtain differential (□CT) expression. Thus □CT values indicate high gene expression.

**Results** *CBFA2* expression was significantly increased in the disease state in all ALL patients ($P < 0.0001$). The expression levels normalized with remission. This difference in the expression was not observed in those AML, where CBFA2 expression remained unchanged with disease status. There was no obvious difference in the *ETV6* expression between disease and remission states for any groups of patients. However, *ETV6* expression was significantly elevated in the diseased state in Group B, as compared to Group A. The three out of four patients with matched pair samples at presentation and at relapse showed decrease in *ETV6-CBFA2* expression, but this is not statistically significant.

**Conclusion** This study shows that *CBFA2* is upregulated in the diseased state of all ALL patients. Since *CBFA2* is involved in acetylating chromatin, histone acetyl transferases may have a therapeutic role in childhood ALL. The decrease in level of *ETV6-CBFA2* expression in the relapsed state, suggests that secondary events are responsible for recurrence of disease.

### 10.5 A UKCCG AND UKCCSG STUDY OF KARYOTYPE DATA FROM PATIENTS WITH EWING'S SARCOMA TUMOURS

P Roberts[1], CA Felix[1], IJ Lewis[2], SA Burchill[3], [1]Dept Cytogenetics, St James's Hospital, Leeds LS9 7TF, [2]Paediatric Oncology & [3]Candlelighter's Research Dept, St James's, Leeds, UK

Prognostic parameters for patients with Ewing's sarcoma (ES) tumours include presence or absence of metastatic disease at diagnosis, tumour volume and site. Over 85% demonstrate a t(11;22) translocation with a further 5% showing a variant of this, creating a gene fusion between EWS at 22q12 and an ETS family gene on the partner chromosome. A wide variety of fusion transcript types have been described, the nature of which has a bearing on prognosis. However, some patients with apparently favourable clinical and molecular genetic features still relapse and die, suggesting other factors may contribute to disease prognosis and progression.

80% of ES patients demonstrate additional secondary chromosome changes at diagnosis, although few studies have assessed the possible clinical importance of these. Our UKCCG and UKCCSG study involves the collection of karyotype data from UK patients aged up to 30 years with ES tumours in order to assess the nature and possible prognostic effect of any consistent secondary chromosome changes.

We have received karyotype data from 105 chromosomally abnormal individuals from 12 UK centres with ES tumours, the largest series to date. 76% demonstrated secondary chromosome changes at diagnosis, typically simple trisomies, the most frequent of which were trisomy 8 (35%) and trisomy 12 (17%). Besides 1p deletion and an unbalanced t(1;16) translocation, there have been few documented reports of secondary structural chromosome imbalance. Our studies revealed 1p36 deletion in 9% and 16q loss in 21%, as well as several previously undocumented deletions – i.e. 3q (10%), 9p (9%), 11q (6%) and 17p (7%).

Initial indications of the data suggest that individuals with complex karyotypes at diagnosis fare worse than those with simple changes, irrespective of metastases. We now intend to assess whether any of the recurrent chromosome abnormalities have prognostic implications, particularly in those individuals where the number of these is relatively low. We hope that further evaluation of these chromosome changes may shed more light on the underlying molecular genetic changes and their contribution to disease progression and survival. We hope to complete the study by early 2002.

### 10.6 GROWTH FACTOR PROFILE OF TUMOURS OF THE EWING'S SARCOMA FAMILY EXAMINED USING CDNA ARRAYS

J Withey and SA Burchill, Candlelighter's Children's Cancer Research Laboratory, ICRF Clinical Centre, St James's University Hospital, Leeds LS9 7TF, UK

Growth factors and their receptors are important in normal cell growth, activating signalling pathways which can stimulate or inhibit cell division, differentiation and migration. Aberrant expression of these proteins can contribute to tumorigenesis by modulating tumour cell attachment, growth and angiogenesis.

We have previously shown that Ewing's Sarcoma (ES) cell lines can maintain their cell number under serum-free conditions and that ES conditioned media provides these cells with a survival advantage compared to normal media (1). The aims of this work were to determine 1) what growth factors/receptors are expressed by these cell lines and tumours which might mediate such an advantage and 2) if the expression of any of these growth factors may be prognostically significant.

Total RNA extracted from 6 ES cell lines and 2 tumour samples taken at diagnosis was labelled and hybridised to cDNA cytokine/receptor arrays (Clontech). Results were analysed using AtlasImage 1.01 (Clontech) which identified seventeen growth factor or receptor genes highly expressed in more than half of the cell lines and both tumours. Four were present in all samples, thymosin beta 10, pleiotrophin, smoothened and p75. The expression of these growth factors was confirmed by reverse transcriptase-polymerase chain reaction (RT-PCR) and Southern blotting or sequencing. Two of the growth factors, tymosin beta 10 and pleiotrophin were explored in greater detail.

Expression of the growth factor pleiotrophin was confirmed at the protein level by immunohistochemistry in tumour sections. The presence of its receptor, syndecan-1, was also identified by RT-PCR and immunohistochemistry. As pleiotrophin is a secreted growth factor, heparin affinity chromatography was used to concentrate conditioned media from ES cell lines and determine whether this growth factor was secreted by the cells.

No antibodies are currently available commercially to the second growth factor, thymosin beta 10. Antibodies were raised in rabbits to the carboxy terminal of this peptide and also isolated from a phage library (NISSIM, MRC) by selecting against the same peptide. These antibodies were used in immunohistochemistry to explore the prognostic significance of thymosin beta 10 in ES.

In summary we have identified 2 growth factors not previously described in ES which may be useful as novel prognostic indicators and/or targets for therapy.

1. Withey J., Burchill S.A. 2000 *Br J Cancer* **83** (Supp 1) 33

### 10.7 BASIC FIBROBLAST GROWTH FACTOR (BFGF)-INDUCED CELL DEATH IN EWING'S SARCOMA IS THROUGH A CASPASE-DEPENDENT AND P53-INDEPENDENT MECHANISM

Westwood G, Burchill SA, Candlelighter's Children's Cancer Research Laboratory, St James's University Hospital, Leeds LS9 7TF, UK

Basic fibroblast growth factor (bFGF) is mitogenic for a number of different cell types and has been implicated in the development and growth of many cancers. However, we have recently shown that bGFG arrests ESFT cells at the $G_1$ checkpoint and induces cell death (Sturla *et al*, 2000). The aim of this study was to identify the mechanism of bFGF-induced cell death in ESFT cells. Using a general caspase inhibitor (Z-VAD-FMK), caspase activity was assessed using the CaspaTag™ flourescein (FAM-VAD-FMK) caspase activity kit (Intergen) as well as the trypan blue exclusion assay. Initiator and effector caspases involved in bFGF-induced cell death were identified using the caspase substrate set III (Calbiochem) and western blotting for cleavage of poly (ADP-ribose) polymerase (PARP). Immunohistochemistry and western blotting measured expression of bcl-2 family members. Mitochondrial transmembrane potential was analysed using tetramethylrhodamine ethyl ester percholate (TMRE) labelling and FACs analysis; cytochrome *c* release was characterised by subcellular fractionation and western blot. p53 gene status in the ESFT cell lines was analysed by single strand conformational polymorphism (SSCP) and sequencing. Caspase activity was first detected 36 h post-bFGF (10 ng/ml) treatment. The caspase inhibitor Z-VAD-FMK (10 μM) significantly protected TTC-466 and TC-32 cells from bFGF-induced cell death ($p<0.01$). The initiator (caspase-2, -8, -10) and effector (caspase-3, -6, -7) caspases were activated in the TC-32 and TTC-466 cells after treatment with bFGF (10 ng/ml) treatment. Expression of bcl-2 was down-regulated and bax up-regulated in TC-32 and TTC-466 cells treated with bFGF (10 ng/ml) for 48 h. Furthermore, high basal expression levels of bcl-2 was observed in the bFGF-unresponsive ESFT cell line (A673), with both bFGF-responsive (TC32, TT466) and unresponsive (A673) ESFT cell lines having mutated p53. In conclusion, bFGF-induced cell death in ESFT is through a caspase-dependent and p53-independent mechanism. Furthermore, bcl-2 may protect ESFT from bFGF-induced cell death.

Sturla L et al. 2000, *Cancer Res* **60:** 6160

### 10.8 DAMAGE-INDUCED BAX N-TERMINAL CHANGE AND TRANSLOCATION TO MITOCHONDRIA OCCUR REGARDLESS OF CELL FATE

G Makin, A Thistlethwaite, B Corfe, G Griffiths, J Hickman[1], C Dive, CRC Molecular and Cellular Pharmacology Group, School of Biological Sciences, University of Manchester, Manchester M13 9PT, UK, [1]Institut de Recherches Servier, Suresnes, 92150, Paris, France

Resistance to chemotherapy is the major obstacle to the successful treatment of neuroblastoma. A model system for the investigation of drug-resistance in vitro is described, exploiting two NB cell lines, SH-EP1 and SH-SY5Y, derived from the same parental background. These subclones show no difference in their sensitivity to the DNA damaging agent cisplatin, but have very different responses to the microtubule stabilising agent paclitaxel; SH-EP1 cells are sensitive, whilst SH-SY5Y cells are resistant. The protein product of the tumour suppressor gene p53 is stabilised to the same extent in SH-SY5Y cells following exposure to cisplatin, which readily engage apoptosis, as in those exposed to paclitaxel, which do not. Stabilised p53 is active in SH-SY5Y cells following paclitaxel exposure as reflected by the transcriptional upregulation of the cyclin dependant kinase inhibitor, $p21^{WAF-1}$, a downstream effector of p53, after both drug treatments. The pro-apoptotic Bcl-2 family protein Bax is latent in healthy cells and requires activation by drug-damage signals. Exposure of an epitope in the N-terminus of Bax was observed in both NB cell lines following both types of drug induced damage. This N-terminal exposure occurred to the same extent in settings of drug resistance as in those of drug sensitivity. The exposure of the N-terminus of Bax occurred in the cytosol, and was followed by the translocation of Bax to the mitochondria, again irrespective of cell fate. The exposure of the N-terminus of Bax was also observed following detachment of NB cells into suspension. Thus the N-terminal changes in Bax represent a reversible response to disparate types of damage, and do not commit the cell to death. A model for the activation of Bax by drug-induced damage in NB cells is suggested that must require a second signal, after N-terminal epitope exposure and mitochondrial translocation, which is needed to commit the cell to apoptosis. This damage-induced second signal is suggested to be abrogated in SH-SY5Y cells after treatment with paclitaxel. Lack of the full activation of Bax may represent a novel method of drug resistance in NB cells.
